# Fluorescence resonance energy transfer (FRET) spatiotemporal mapping of atypical P38 reveals an endosomal and cytosolic spatial bias

**DOI:** 10.1038/s41598-023-33953-y

**Published:** 2023-05-08

**Authors:** Jeremy C. Burton, Jennifer Okalova, Neil J. Grimsey

**Affiliations:** 1 Department of Pharmaceutical and Biomedical Sciences, College of Pharmacy, University of Georgia, Pharmacy South Rm 414, Athens, 30602 USA; 2 Present Address: Aflac Cancer and Blood Disorders Center, Department of Pediatrics, School of Medicine, Emory University, Atlanta, GA 30322 USA

**Keywords:** Cellular imaging, Cell biology, Cell signalling, Hormone receptors, Stress signalling

## Abstract

Mitogen-activated protein kinase (MAPK) p38 is a central regulator of intracellular signaling, driving physiological and pathological pathways. With over 150 downstream targets, it is predicted that spatial positioning and the availability of cofactors and substrates determines kinase signaling specificity. The subcellular localization of p38 is highly dynamic to facilitate the selective activation of spatially restricted substrates. However, the spatial dynamics of atypical p38 inflammatory signaling are understudied. We utilized subcellular targeted fluorescence resonance energy transfer (FRET) p38 activity biosensors to map the spatial profile of kinase activity. Through comparative analysis of plasma membrane, cytosolic, nuclear, and endosomal compartments, we confirm a characteristic profile of nuclear bias for mitogen-activated kinase kinase 3/6 (MKK3/6) dependent p38 activation. Conversely, atypical p38 activation via thrombin-mediated protease-activated receptor 1 (PAR1) activity led to enhanced p38 activity at the endosome and cytosol, limiting nuclear p38 activity, a profile conserved for prostaglandin E2 activation of p38. Conversely, perturbation of receptor endocytosis led to spatiotemporal switching of thrombin signaling, reducing endosomal and cytosolic p38 activity and increasing nuclear activity. The data presented reveal the spatiotemporal dynamics of p38 activity and provide critical insight into how atypical p38 signaling drives differential signaling responses through spatial sequestration of kinase activity.

## Introduction

The highly conserved subfamily of MAPK p38 (consisting of four members α, β, γ, δ) are essential regulators of intracellular stress responses. Dual phosphorylation of p38 at the conserved Threonine-Glycine-Tyrosine (TGY) motif (T180 and Y182) by the upstream MAP kinase kinase (MKK3/6) is essential for classical p38 activation. MKK3/6 can activate all four family members, regulating many physiological and pathological processes, including cell growth, proliferation, wound healing, and inflammation^1^. The diverse physiological functions and the conserved mechanism of activation by MKK3/6 dictates that intracellular signaling is regulated through substrate availability and intracellular localization of p38.

Cellular stress induces p38 activation and intracellular redistribution, targeting p38 to selective pools of substrates. In a resting state, p38 is typically distributed between the cytosol and nucleus. However, after activation, all p38 isoforms are rapidly translocated to the nucleus, a step that is critical for nuclear resident transcription factor activation^2^. Conversely, p38 also has critical roles in the cytosol, activating cytosolic transcription factors to induce their nuclear translocation and activating other cytosolic kinases^2,3^.

**Figure 1:**
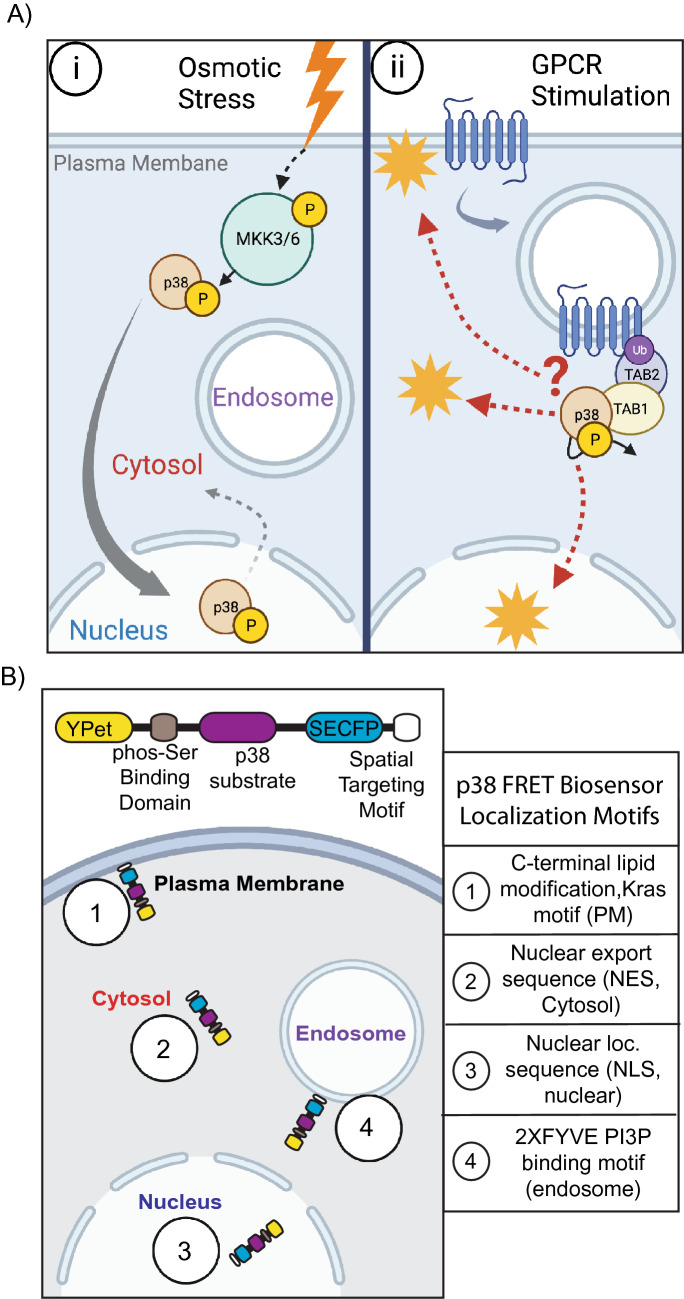
Model of p38 activation, localization, and translocation. (**A**) (i) Schematic representation of cellular stress-induced MKK3/6 activation and translocation of p38 cohorts. Rapid translocation to the nucleus and recycling back into the cytosol (ii) Atypical p38 activation after GPCR stimulation, unclear spatiotemporal dynamics (**B**) Schematic representation of spatially targeted p38 FRET biosensors, C-terminal fused subcellular localization motifs (1) plasma membrane, (2) cytosol, (3) nucleus, (4) endosome.

In addition to the classical MKK3/6 activation pathway (Fig. 1Ai), p38 can also be activated by two selective atypical mechanisms that direct p38 autophosphorylation and activation^3^. The first selectively occurs in T-cells, utilizing the tyrosine kinase ZAP70 to phosphorylate p38 at Tyr323. Phospho-Tyr323 triggers a conformational change driving p38 autophosphorylation at Thr180 in *trans* between homo or heterodimers of p38α and p38β^4,5^. The second atypical p38 pathway is mediated by the direct binding of p38 to the adaptor protein transforming growth factor beta-activated-kinase-1-binding protein-1 (TAB1)^6^. The c-terminal peptide of TAB1 selectively binds only the p38α isoform at two distinct binding sites, inducing a conformational change and enabling p38α autophosphorylation^8,9^. Critically, TAB1-dependent atypical p38 activity has been shown to regulate key pathological processes in a widening array of diseases, including cardiac damage, vascular inflammation, viral and parasite infections, recently reviewed^3^.

Atypical p38 is initiated through several mechanisms, including hypoxia, oxidative stress, and G protein-coupled receptors (GPCRs). Stimulation of multiple inflammatory GPCRs can trigger activation of an E3 ubiquitin ligase, NEDD4.2, through a conserved tyrosine phosphorylation switch mechanism, inducing GPCR ubiquitination and scaffolding of the atypical p38 signaling complex through TAB2 binding to ubiquitinated GPCRs and recruitment of TAB1^11–11^ (Fig. 1Aii). Nevertheless, the explicit mechanisms that control TAB1-p38-dependent atypical activity through GPCR-independent pathways have not been described in detail^3,6,8,10^. Regardless, there are several common factors during atypical signaling: the TAB1-p38 interaction is relatively stable^8^, atypical p38 activation induces TAB1 phosphorylation, TAB1 phosphorylation blocks TAB1 interaction with TAK1 further amplifying the atypical pathway^12^, and the dual phosphorylation of p38 is identical to MKK3/6-dependent phosphorylation (T180 and Y182)^6^. Atypical p38 signaling must therefore exhort differential cellular responses through an alternative regulatory process.

Recent studies have highlighted the spatiotemporal control of G-proteins and MAPKs at the endosome, Golgi, and ER, driving selective and physiologically critical signaling responses where the subcellular location of the kinases or receptors induces selective cellular responses^15–16^. Advances in genetically encoded Fluorescence Resonance Energy Transfer (FRET)-biosensors have enabled detailed spatial studies of ERK1/2 activity using the EKAR4 ERK activity biosensors^17^. Despite the development of a comparable p38 activity FRET biosensor^18^ there have been no studies into the spatial control of atypical p38 MAPK.

This study aimed to develop a platform of subcellular targeted genetically encoded FRET biosensors to spatially map p38 activity. Using live-cell imaging of cells transfected with p38 FRET sensors targeted to the plasma membrane, cytosol, nucleus, and endosome, (Fig. 1B), we compared the kinase activity between osmotic stress and thrombin-mediated activation of protease-activated receptor 1, PAR1. We are the first to map the spatial dynamics of p38 activity in live cells and demonstrate a differential spatiotemporal profile for atypical p38 signaling, biasing p38 to the endosome and cytosol. These studies provide critical insight into how atypical p38 drives differential pathological inflammatory signaling during vascular inflammation and establish a broader paradigm for understanding atypical p38 responses.

## Results

### Spatial targeting of p38 FRET sensors

**Figure 2:**
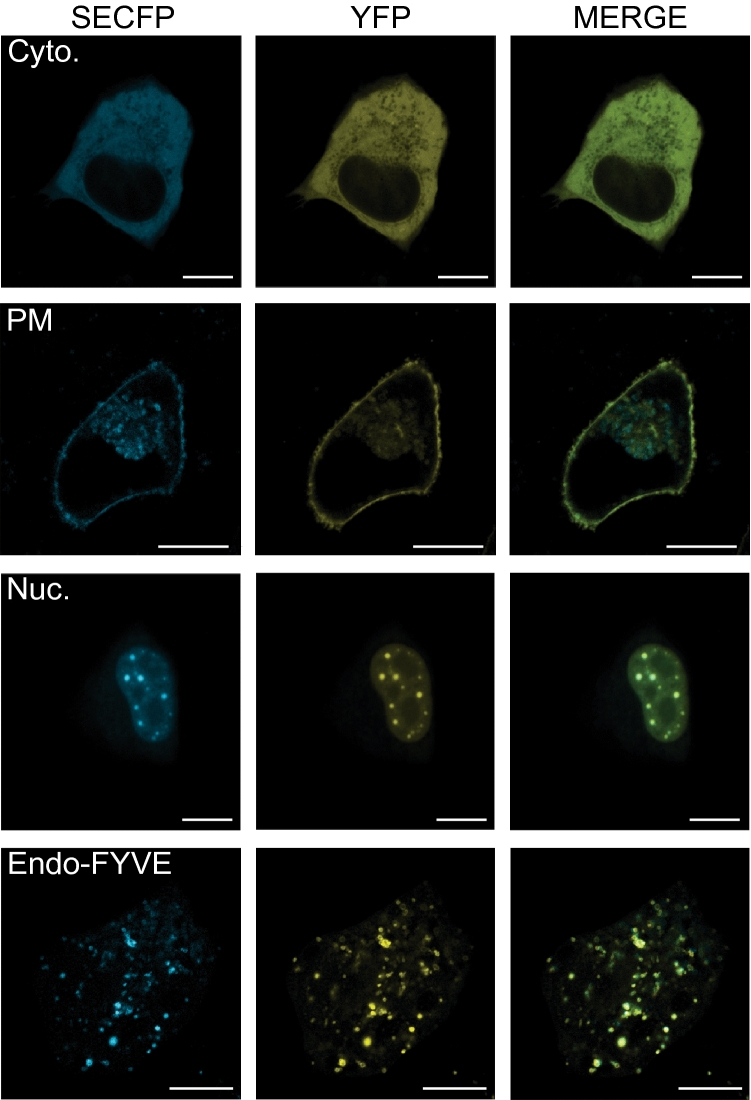
P38 biosensor platform localized to subcellular compartments. (**A**) Representative fluorescent (SECFP, YFP, merged) confocal images of HeLa cells transiently expressing biosensors with spatial targeting motifs for the plasma membrane (PM), cytosol (Cyto.), nucleus (Nuc.), or endosome (Endo-FYVE). Scale bars, 10 µm.

Recent studies described using a p38 FRET sensor to examine MKK3/6-dependent p38 oscillations in HeLa cells^18^. The FRET biosensor is a single molecule, with an n-terminal YFP and c-terminal SECFP, linked by a flexible peptide containing a p38 binding and phosphorylation motif and a WW domain (Fig. 1B)^18^ We proposed that the addition of subcellular targeting motifs to this previously characterized activity biosensor would enable spatiotemporal mapping of MKK3/6 and TAB1-dependent p38 activation pathways (Fig. 1A). To determine the activity of p38 at distinct intracellular sites of the cell, we generated c-terminal tagged p38 FRET with a KRAS (Kirsten Rat Sarcoma viral oncogene homolog)-motif to target the plasma membrane (PM)^19^, the 2× FYVE (Fab1, YOTB/ZK632.12, Vac1, and EEA1, zinc finger) domains from hepatocyte growth factor-regulated tyrosine kinase substrate (HRS) to target the endosome via binding to the membrane lipid phosphatidylinositol 3-phosphate, PtdIns3*P* which are enriched in early endosomes^20,21^, a nuclear export sequence (NES, cytosol) to target the cytosol^22^, and nuclear localization sequence (NLS, nuclear) to target the nucleus^23^ (Fig. 1B). The spatial p38 FRET sensors were transiently expressed in HeLa cells, and confocal imaging revealed the predicted localizations of each biosensor (Fig. 2A), assessed via costaining with specific spatial markers and calculating Pearson correlation coefficients (*r*); Biotracker™ orange = plasma membrane, *r* = 0.466 ± 0.131; GAPDH-647 = cytosol, *r* = 0.351 ± 0.107; RedDot^TM^2-far-red nuclear stain = nucleus, *r* = 0.436 ± 0.132; (Suppl. Fig. 1A–C). The subcellular localization of the 2xFYVE endosomal sensor was additionally validated by coexpression with the endosomal associated wild type GTPase Rab5 (Rab5-mRFP), or constitutively active Rab5 Q79L-mRFP, which displays an endosomal trafficking and fusion defect leading to enlarged endosomes^24,25^. Pearson correlation coefficients (*r*) (wt Rab5 *r* = 0.448 ± 0.13, or Q79L Rab5 *r* = 0.446 ± 0.15) indicated that 2xFYVE targeting of the biosensor colocalized with Rab5 positive early endosomes (Suppl. Fig. 1D,E), as previously shown^26–26^.

### Osmotic stress by NaCl induces rapid p38 activation with the peak in the nucleus

**Figure 3:**
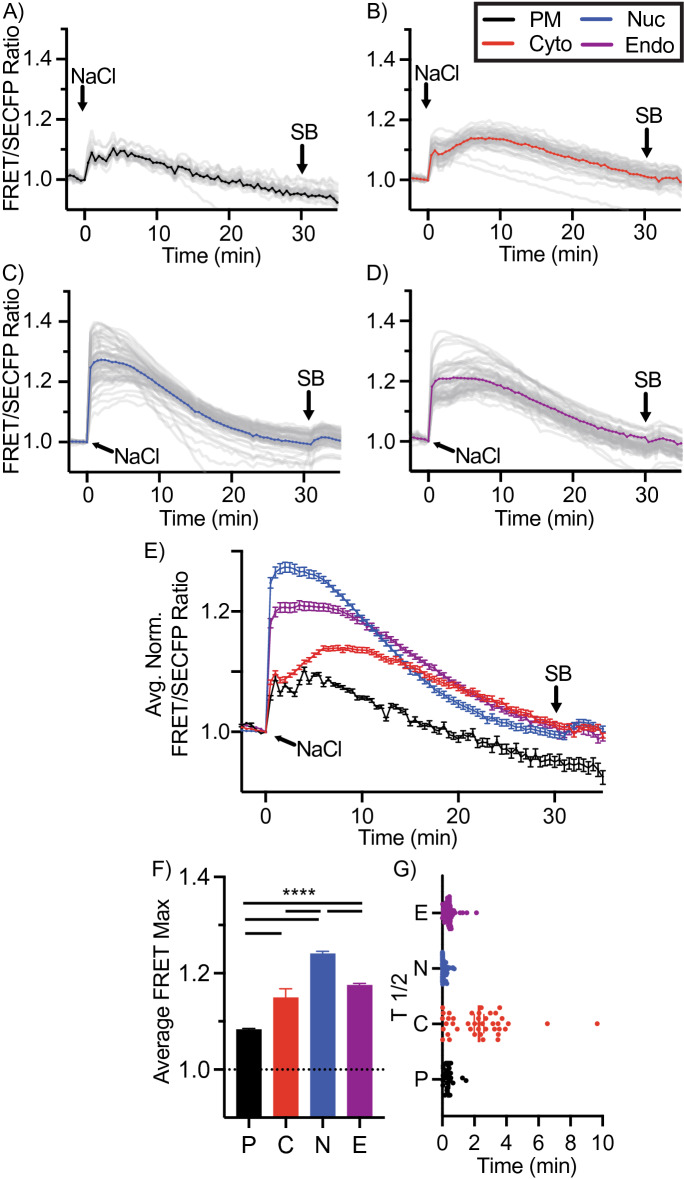
Subcellular mapping of MKK3/6-dependent p38 activity. (**A**–**D**) Activation of p38 using FRET biosensors localized to the plasma membrane (**A**), cytosolic (**B**), nuclear (**C**), or endosomal (**D**) expressed in HeLa cells incubated with 300 mM NaCl and SB203580 (SB). FRET ratios were normalized prior to NaCl addition. Individual cell FRET ratios are depicted in gray (n > 30–60 cells per biosensor). Average FRET ratio is indicated by colored lines. (**E**) Overlay of representative normalized averages of FRET ratio in biosensor platform from (**A**–**D**). (**F**) Maximum change in FRET ratio (FRET_MAX_) of individual cells pooled from 3 independent repeats, (n > 80–150 ROIs per biosensor. mean ± SEM) were analyzed by One-Way ANOVA (****p < 0.0001). (**G**) FRET response kinetics, T ½ values of cells in (**A**–**D**) (mean ± SEM).

To validate the platform of spatial biosensors, we first utilized NaCl-induced osmotic shock. Osmotic shock is a classic example of MKK3/6 p38 activation, where RAC GTPase interacts with and activates an osmosensing complex driving MEKK3-dependent MKK3 activation. MKK3-dependent p38 phosphorylation induces a rapid translocation of p38 to the nucleus^27^. Osmotic stress was induced using 300 mM NaCl in cells expressing the p38 sensor panel. Baseline FRET responses were allowed to stabilize before stress induction. Osmotic stress induced rapid p38 activity in all four spatial constructs, with p38 activity measured as normalized FRET/SECFP ratio over time, the grey lines representing individual cells, and the color line representing the experimental average (Fig. 3A–E), representative examples for ratiometric FRET responses displayed as pseudo color look up table (LUT) images (Suppl. Fig. 2). Maximal FRET response (FRET_MAX_) was rapidly achieved with a FRET_T½_ (half the time to reach FRET_MAX_) within 30 s in all but the cytosol, in which individual cells had a greater distribution of FRET_T½_ values over 2–4 min with an average of 2 min (Fig. 3F,G). The PM and cytosolic biosensor FRET_MAX_ peaked at 1.08 (black) and 1.14 (red) over baseline, respectively (Fig. 3A,B,E,F). The endosomal population peaked at 1.17, which was not significantly different from the cytosolic signal. Conversely, the nuclear FRET_MAX_ peaked at 1.24 over baseline, significantly higher than the other populations (Fig. 3C,E,F, blue). Note that NaCl induced p38 activity returned close to basal levels at 30 min and SB203580 addition displayed no further reduction in FRET responses, Fig. 3A–E as indicated).

### Spatial mapping of thrombin activation of PAR1 reveals a differential atypical p38 profile

**Figure 4:**
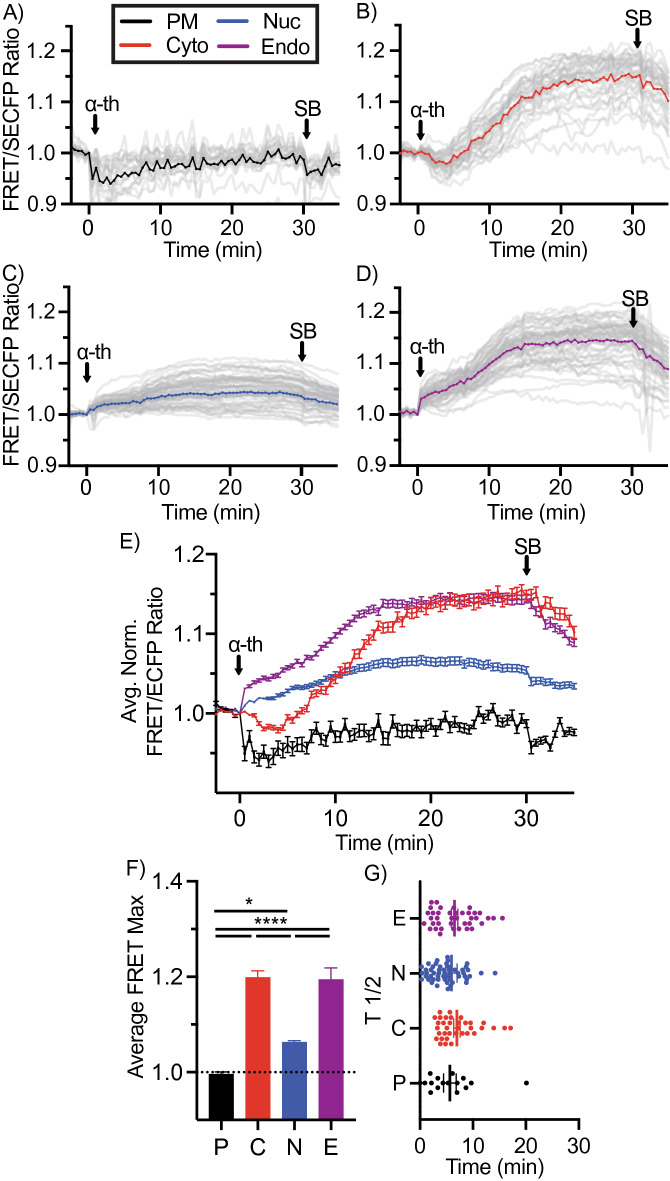
P38 activation is differentially regulated by thrombin. (**A**–**D**) Activation of p38 using FRET biosensors localized to the plasma membrane (**A**), cytosolic (**B**), nuclear (**C**), or endosomal (**D**) expressed in HeLa cells incubated with 10 nM thrombin (α-Th) and SB203580 (SB). FRET ratios were normalized prior to thrombin addition. Individual cell FRET ratios are depicted in gray (n > 30–60 cells per biosensor). Average FRET ratio is indicated by colored lines. (**E**) Overlay of representative normalized averages of FRET ratio in biosensor platform from (**A**–**D**). (**F**) Maximum change in FRET ratio (FRET_MAX_) of individual cells pooled from 3 independent repeats, (n > 80–150 ROIs per biosensor. mean ± SEM) were analyzed by One-Way ANOVA (*p < 0.05, ****p < 0.0001). (**G**) FRET response kinetics, T ½ values of cells in A-D (mean ± SEM).

We have previously established that the thrombin activated GPCR, PAR1, induces a robust atypical p38 activation pathway^9,11^. To determine whether thrombin activation of PAR1 induced spatially selective atypical p38 dynamics we co-transfected HeLa cells with PAR1 and each of the four biosensors. Thrombin-induced robust p38 activity in the cytosol, peaking with a FRET_MAX_ of 1.2 (Fig. 4B,E,F, red, and pseudo color LUT images Suppl. Fig. 3A). A reduction of p38 FRET consistently preceded the increase in p38 activity immediately after thrombin stimulation. This transient drop of p38 cytosolic activity lasted ~ 5 min before increasing. Notably, thrombin displayed slower p38 activation dynamics with a FRET_T1/2_ of ~ 8 min compared to osmotic stress (Figs. 3G and 4G). Contrary to the cytosolic signal, thrombin induced a rapid initial increase in p38 activity at the endosome. Within 1 min, the FRET ratio reached ~ 1.05, before a slower gradual rise to a FRET_MAX_ of 1.19. The FRET_MAX_ was comparable for the endosomal and the cytosolic sensors (Fig. 4B,D,E, pseudo color LUT images Suppl. Fig. 3B, compared to NaCl treatment Fig. 3B,D,E, red and purple). Contrary to the transient activation of the p38 FRET responses after osmotic shock, thrombin activity was sustained. To confirm that the FRET sensor was not locked in active confirmation, endosomal and cytosolic FRET activity were blocked at 30 min using the p38 selective chemical inhibitor, SB203580^28^ (Fig. 4A–E as indicated), but not after DMSO addition (Suppl. Fig. 4A–D as indicated) demonstrating that the detected increase in FRET ratios and SB203580 dependent loss are due to p38 activity. The endosome is hypothesized to be a central nexus of PAR1 p38 signaling^9,11,29^. To confirm the rapid endosomal signaling by thrombin, we targeted the FRET biosensor to the endosome by replacing the small 2xFYVE PtdIns3*P* binding domain with the larger full-length early endosomal GTPase, Rab5^20^, on the c-terminus of the FRET biosensor. The Rab5-directed sensor colocalized with the early endosomal associated protein 1, EEA1 (Suppl. Fig. 1) and also displayed a rapid thrombin-mediated activation reaching a FRET_MAX_ of 1.09 at 3 min before dipping and rising again (pseudo color LUT images Suppl. Fig. 3E, Suppl. Fig. 5A purple). However, contrary to the 2xFYVE sensor, NaCl failed to solicit a robust Rab5-FRET response (pseudo color LUT images Suppl. Fig. 2E, Suppl. Fig. 5B).

Unlike the rapid nuclear FRET response seen for NaCl, thrombin induced a minimal but consistent nuclear signal, with a FRET_MAX_ peaking at ~ 1.06 (Figs. 3C,E, 4C,E blue, pseudo color LUT image Suppl. Fig. 3C).

While NaCl induced a rapid increase in p38 activity at the plasma membrane (Fig. 3A,E black), thrombin-induced plasma membrane responses were distinctly different, as FRET ratios rapidly dropped below baseline to ~ 0.95 before slowly recovering, but never rising above baseline FRET signal (Fig. 4A,E black, pseudo color LUT image Suppl. Fig. 3C). Of note, all acquisition parameters were identical to those during NaCl treatment. SB203580 addition post-stimulation had no detectable influence on the thrombin induced PM-FRET activity (Fig. 4A, E black).

While GPCR-induced p38 activity was detected in the endosome and cytosol, thrombin-mediated activation of p38 displayed a distinctly differential profile to that seen for osmotic stress in Fig. 3, biasing p38 activity away from the nucleus into the cytosol and endosome (Fig. 4). We have previously shown that atypical p38 signaling is conserved for a family of inflammatory GPCRs. To confirm the GPCR-specific spatiotemporal bias for p38 activity, we examined prostaglandin activation of the prostaglandin E receptors 2 and 4, EP2 (PTGER2) and EP4 (PTGER4). Both receptors displayed comparable spatial profiles to that seen for thrombin-activated PAR1, with peak activations for cytosolic and endosomal sensors and minimal activation at the PM or in the nucleus (Suppl. Figs. 6A–F (EP2) and 7A–F (EP4)), although cytosolic activation was slower for both receptors than seen for thrombin (Suppl. Figs. 6G (EP2) and 7G (EP4)).

### FRET response is driven by p38 activity

**Figure 5:**
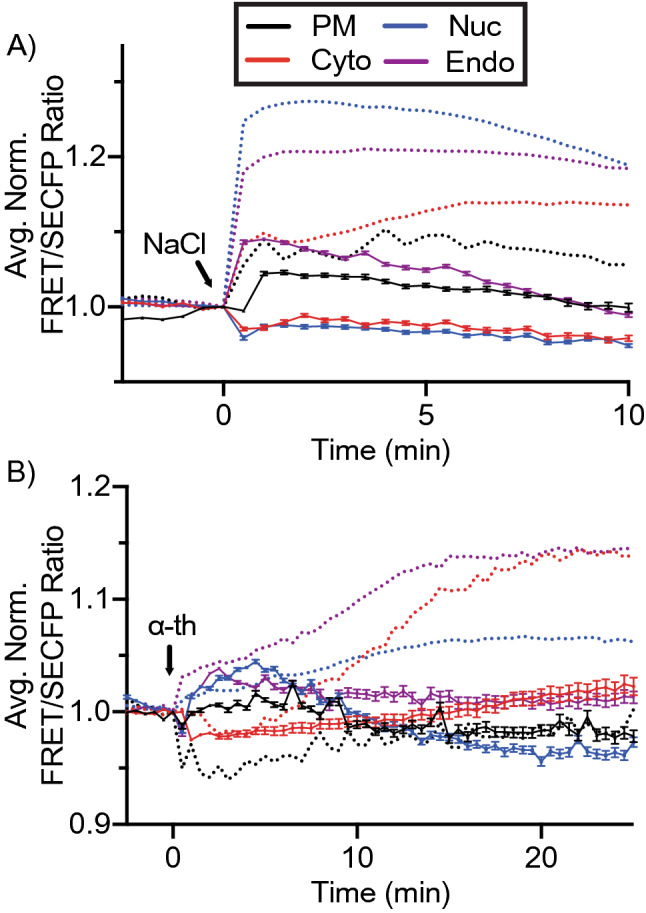
FRET response driven by p38 activity. Activation of p38 using FRET biosensors localized to the plasma membrane, cytosolic, nuclear, or endosomal expressed in HeLa cells preincubated with SB203580 (SB) prior to 300 mM NaCl (**A**) or 10 nM thrombin (α-Th) (**B**). FRET ratios were normalized prior to the addition of NaCl or thrombin. The average FRET ratio is indicated by solid-colored lines, dashed lines indicate representative plots from Fig. 3 in A and Fig. 4 and B, and representative plots from > 2 independent repeats (n > 30–60 cells ROIs per biosensor. mean ± SEM).

The p38 biosensor was previously shown to be selective for p38α in response to interleukin 1β^18^. We show in Fig. 4A–D that the addition of SB203580 suppresses FRET responses in the cytosolic, endosomal, and nuclear compartments, but as thrombin did not induce activity at the plasma membrane, the inhibitor did not reduce this FRET value further. To confirm that the detected FRET response for GPCR signaling is specific to p38 activity, cells were preincubated with SB203580 before stimulation with thrombin or NaCl. All FRET sensors displayed reduced FRET_MAX_ compared to values shown in Figs. 3 and 4 (combined in Fig. 5). SB203580 blocked thrombin responses in cytosolic, endosomal, and nuclear FRET ratios. Notably, the drop in PM FRET ratio in response to thrombin (Fig. 4A) was blocked by SB203580 pretreatment, with the FRET ratio remaining close to the baseline and comparable to cytosolic, endosomal and nuclear biosensors pretreated with SB203580 (Fig. 5B, compare black dotted line with black full line). SB203580 preincubation also suppressed Rab5 FRET responses (Suppl. Fig. 5C,D). To further confirm the specificity of the p38 FRET sensor we also generated a null-FRET-cytosolic sensor, mutating the critical p38 target serine residues to alanine (Suppl. Fig. 8A). The cytosolic null-FRET blocked both thrombin (Suppl. Fig. 8B) and NaCl (Suppl. Fig. 8C) responses.

### Inhibition of receptor internalization spatially flips GPCR signaling dynamics

**Figure 6:**
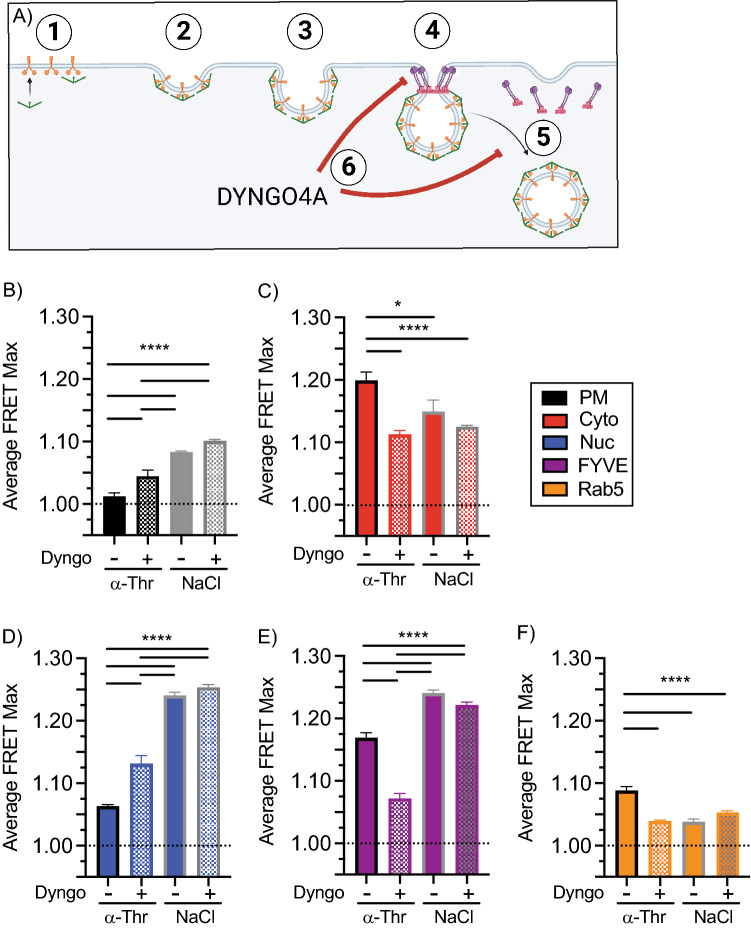
Blockade of GPCR internalization changes spatial FRET bias. HeLa cells transiently expressing biosensors and PAR1 were preincubated with 15 µM Dyngo4A for 45 min prior to stimulation with 10 nM thrombin (α-Th) or 300 mM NaCl. (**A**) Schematic representation of clathrin-mediated endocytosis and the site of action of Dyngo4A inhibition, (1–3) cargo and adaptor proteins drive the formation and stabilization of clathrin coated pits on the plasma membrane, (4) dynamin is recruited to the clathrin coated pit (5) with assistance from actin dynamin drives scission and coated vesicle release, (6) Dyngo4A block dynamin-dependent scission events. (**B**–**F**) Average F-max values from Figs. 3 and 4 were compared to respective Dyngo4A F-max grouped by expression of (**B**) plasma membrane (black), (**C**) cytosol (red), (**D**) nucleus (blue), (**E**) endosome (FYVE) (purple) or (**F**) endosome (Rab5) (orange). Data (mean ± SEM) analyzed by one-way ANOVA (****p < 0.0001).

After thrombin activation, PAR1 is rapidly removed from the plasma membrane via clathrin-mediated endocytosis (CME)^32–32^. Prior studies demonstrated that PAR1 ubiquitination and activation of p38 signaling is retained when CME is blocked via the dynamin inhibitor Dyngo4A (Fig. 6A)^11^. This suggests that atypical p38 activity is initiated and sustained at the plasma membrane when CME is inhibited. To examine whether endosomal trafficking is required to drive the spatial bias GPCR-induced signaling to the cytosol/endosome, we treated cells with Dyngo4A before activation.

Initially, we assessed the effect of Dyngo4A pretreatment on osmotic stress-induced p38 activation. Pretreatment of cells with Dyngo4A had a minimal impact on NaCl-induced p38 activation profiles of all four biosensors (Fig. 6B–F, and Suppl. Fig. 9A–F) FRET_MAX_ values from Fig. 3F, solid bars plotted next to Dyngo4A FRET_MAX_ checkered bars). NaCl activity analysis of the FRET_MAX_ for all sensors revealed no significant change (Fig. 6B–F). Dyngo4A also did not change the FRET_T1/2_ (Suppl. Fig. 9G), demonstrating that Dyngo4A does not alter osmotic stress (MKK3/6)- dependent p38 activation, confirmed by immunoblotting (Suppl. Fig. 11A,B, lanes 1–6).

Contrary to NaCl treatment, Dyngo4A treatment was predicted to enhance thrombin-induced PM p38 signaling as CME traps active PAR1 at the PM, recruiting NEDD4-2 to the PM^11^. Dyngo4A pretreatment blocked thrombin-induced loss of FRET signaling at the PM and displayed a small but significant thrombin-dependent increase relative to controls (Fig. 6B, FRET_MAX_ values from Fig. 4F, solid bars plotted next to Dyngo4A FRET_MAX_ checkered bars, Suppl. Fig. 10A,F black). However, FRET_MAX_ for both endosomal and cytosolic biosensors were significantly reduced after inhibition of CME (Fig. 6C,E, FRET_MAX_ values from Fig. 4F, solid bars plotted next to Dyngo4A FRET_MAX_ checkered bars from Suppl. Fig. 10B,D,E,F, red and purple). Rab5 endosomal signaling was also blocked by pretreatment with Dyngo4A (Fig. 6F and Suppl. Fig. 12A,B). Contrary to the cytosol and endosome, the nuclear p38 displayed a significant increase in FRET_MAX_ of 1.13 (Fig. 6D blue, Suppl. Fig. 10C,E,F blue). Despite these changes in spatial FRET signaling, p38 phosphorylation was unaffected by Dyngo4A (Suppl. Fig. 11A,B, lanes 7–12).

**Figure 7:**
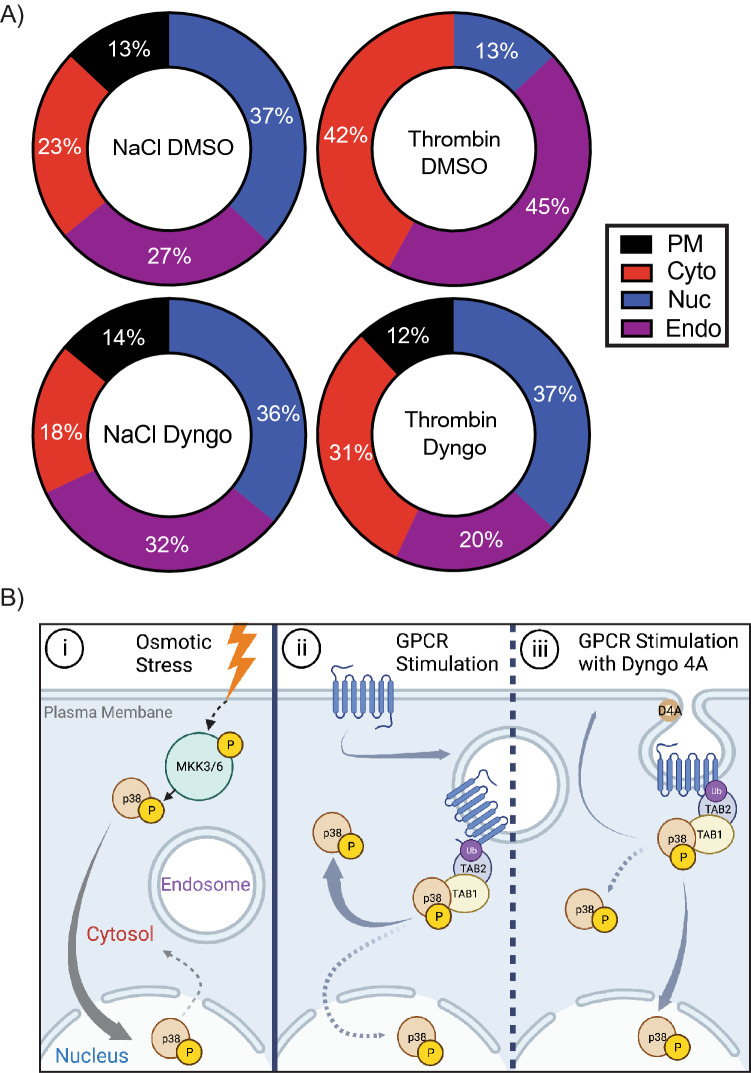
Atypical p38 signaling model. (**A**) FRET max values from each biosensor previously calculated in Figs. 3, 4, Suppl. S6, S7, compiled as a percentage of the total FRET_MAX_ responses. (**B**) Proposed schematic of p38 spatial localization. (i) osmotic stress as described in Fig. 1. (ii) GPCR-mediated TAB1-p38 activation is rapidly trafficked off the plasma membrane to the endosome and the cytosol, with a small portion of nuclear p38. (iii) GPCR-mediated stimulation with Dyngo4A preincubation arrests internalization and reduces p38 activity in the cytosol and endosome, increasing p38 trafficking to the nucleus.

When correlating the contribution of each compartment to the collective maximal FRET signal for all locations, the relative ratios of NaCl FRET responses were unchanged after the addition of Dyngo4A. Nuclear p38 represented 37%, endosome 27% cytosol 23% and plasma membrane 13% (Fig. 7A), whereas thrombin displayed 45% endosome, 42% cytosol, and 13% nuclear signaling. After Dyngo4A treatment, the ratios switched for thrombin activation, with 12% at the plasma membrane, 20% endosome, 31% cytosolic, and 37% nuclear. Intriguingly the Dyngo4A flipped the distribution of p38 activation (Fig. 7A,B) so that it mirrored the distribution seen after NaCl treatment (Fig. 7A,Biii). Together these data suggest that atypical p38 signaling is spatially biased to the cytosol/endosome and that blockade of receptor internalization can flip the spatial bias to mimic that seen for MKK3/6-dependent signaling driving p38 activity into the nucleus (Fig. 7Biii).

## Discussion

Spatial regulation of kinases is critical for determining downstream functional outcomes. While there have been extensive studies into the spatial control of MKK3/6-dependent p38 activation, there is a limited understanding of the spatial kinetics of atypical p38 activation, specifically after GPCR activation. In this study, we utilized a platform of spatially targeted p38 FRET biosensors to conduct live-cell imaging experiments to investigate the spatiotemporal landscape of p38 activation in response to osmotic stress or thrombin-dependent GPCR activation. We demonstrate that GPCR-induced TAB1-p38 signaling displays a differential temporal profile of activity. Contrary to MKK3/6-dependent signaling, TAB1-mediated atypical p38 perturbs nuclear translocation of p38, sequestering and biasing kinase action to the cytosol and endosome (Fig. 7B). We have previously hypothesized that GPCR endocytic trafficking plays a critical role in this spatial bias. However, we now show that the endosomal spatial bias can be switched through inhibition of clathrin-mediated endocytosis, flipping p38 activity to a nuclear bias.

Initially discovered in 2002, atypical p38 activation is mediated by the direct interaction between TAB1 and p38. TAB1 binding drives a critical conformational change in p38, enabling p38 autophosphorylation independent from the classical MKK3/6 mediated activation^6,7^. GPCRs drive atypical p38 activation via a ubiquitin-dependent scaffold, initiating the formation of a critical TAB2-TAB1-p38 signaling complex^3,9,29^. Ubiquitin-dependent initiation of atypical p38 signaling is a conserved mechanism for a family of GPCRs in both macrovascular and microvascular beds^10^.

Despite the predictions regarding the sequestration of TAB1-p38 in the cytosol, prior studies have relied upon fixed time points and overexpression studies to explore nuclear translocation and stabilization of the TAB1-p38 complex^6,8,12,35–37^. While these studies are highly suggestive, there are advantages to live-cell imaging, which allows for a comprehensive assessment of the spatially guided signaling dynamics in individual cells. Recent studies have highlighted this potential by exploring intracellular G-protein dynamics, and spatially resolved ERK1/2 signaling^15–16^, PKA^38^, and AMPK nuclear signaling^39^, defining the critical spatial control of kinases, where adaptor and substrate accessibility drives their functional roles. While it is well known that p38 has a central role in the regulation of both cytosolic and nuclear proteins^2,3^, direct analysis of the spatiotemporal p38 dynamics has not been carried out.

To overcome the lack of tools to explore p38 spatiotemporal signaling dynamics, our current study compared the spatial kinetics of p38 activation at the plasma membrane, cytosol, endosome, and nucleus. As expected, osmotic stress-induced rapid activation at the PM and almost simultaneous translocation to the nucleus, where p38 activity peaked within seconds of activation. This is consistent with prior biochemical studies, as recently reviewed^2^**.**

On the contrary, GPCR activation displayed a distinctly different activation profile, with limited activity in the nucleus and a loss of activation at the PM. This is consistent with prior studies indicating PAR1 is rapidly trafficked away from the plasma membrane through clathrin-mediated endocytosis, CME^11,31,32,40^. Although the E3 ubiquitin ligase NEDD4.2 is critical for atypical p38 activation by GPCRs, blockade of CME using Dyngo4A did not suppress receptor ubiquitination or p38 activation, which suggested that the atypical p38 signaling can occur at the PM. We had predicted that Dyngo4A treatment would lock atypical p38 at the PM, mirroring NaCl signaling dynamics. However, Dyngo4A failed to induce a rapid increase of p38 signaling at the PM, suggesting that either the association with ubiquitinated PAR1 is so rapid that it is undetectable or CME inhibition changes p38 activity by restricting access to the endosome.

Indeed, the temporal dynamics of GPCR CME and endosomal trafficking of the ubiquitinated receptors suggested that atypical p38 signaling would be initiated at the endosome. Prior studies showed ubiquitinated PAR1 rapidly recruits TAB2 to endosomes via a Zinc finger ubiquitin-binding motif (ZnF)^9^. The endosome-targeted FRET sensor showed the fastest p38 activity after thrombin treatment, rapidly responding within 1 min of activation, followed by a later second phase of activation increasing further. Cytosolic p38 activation by thrombin displayed an unexpected initial signal loss, which then steadily increased to match endosomal FRET_MAX_ after 25 min of stimulation. It is tempting to hypothesize that the initial loss of GPCR-induced cytosolic p38 stems from the rapid recruitment of p38 to endosomes.

The cytosolic redistribution was conserved with the prostaglandin receptors EP2 and EP4 but not seen for NaCl treatment which displayed a two-phase increase in p38 activity, an initial rapid activation, followed by a slower rise to FRET_MAX_ at ~ 7 min. We have previously shown that PGE2 activation induces atypical p38 activity, and our data demonstrates that atypical p38 signaling drives a comparable spatial bias for PGE2-induced p38 signaling by the human EP2 and EP4 receptors (PTGER2 and 4, respectively). Further studies will be required to explore how conserved this bias is across GPCRs and non-GPCR-induced atypical p38 signaling.

Of note, NaCl stimulation induced rapid endosomal signaling, suggesting that the endosome may also play a role in the initial phases of osmotic stress-mediated activation of p38.

In addition to the PI(3)P binding 2xFYVE^20^ early endosomal targeted FRET sensor, we also included an alternative endosomal targeting strategy using the full-length wt-Rab5-FRET sensor. Rab5 is an early endosome GTPase that plays a critical role in endosomal trafficking and is classically used as a marker for the endosome^41,42^, Rab5 binds to and colocalizes with EEA1 at PI3P lipid membranes^20^. Differing from the FYVE targeting, Rab5-FRET displayed a rapid peak in p38 activation after thrombin signaling before dipping and rising again. However, NaCl failed to activate Rab5-FRET biosensor responses. As Rab5 is being used as a targeting motif and the GTPase activity is not explicitly being measured, it is possible that the full-length Rab5 could be acting as a steric hindrance displacing the FRET sensors too far from the endosome to detect NaCl-dependent signaling. Conversely, atypical p38 is scaffolded by GPCR-ubiquitin chains that drive the formation of the TAB2-TAB1 signaling complex, and this additional structure may position p38 activity to ideally activate the Rab5-FRET complex. Additional studies will be required to explore this in more detail.

The endosome is essential in the regulation of signaling dynamics for multiple pathways, including for opioid receptors^43^, follicle-stimulating hormone (FSH)^44^, and the beta2-adrenergic receptor (β2-AR)-dependent endosomal signaling^15,16^. Each case alters the specific interactions with molecular adaptors that drive kinase activity. While Dyngo4A treatment didn’t induce the predicted rapid increase in PM p38 signaling, inhibition of CME significantly reduced thrombin-dependent FRET_MAX_ of both endosomal and cytosolic p38 activity (Fig. 7A). Interestingly, in this context thrombin-dependent p38 displayed a temporal map simar to NaCl signaling with a rapid transient peak, albeit activated at a lower intensity than NaCl. Furthermore, Dyngo4A had the most pronounced effect on nuclear FRET activity after thrombin stimulation, inducing a rapid transient nuclear FRET response, with an average FRET_MAX_ double that of the control cells. But, again, Dyngo4A did not affect NaCl-treated nuclear FRET responses. This suggests that the spatial bias of GPCR-induced atypical p38 signaling at the endosome and cytosol (Fig. 7Bii) specifically requires clathrin-mediated endocytosis or at least passage through the endosome. Disruption of this critical step flips the spatial signaling so that the atypical p38 dynamics mimic NaCl-dependent MKK3/6 profile (Fig. 7A,Biii).

The substrate and adaptor accessibility of p38 dynamically controls cellular functions, including the regulation of transcription factors^2,45^, or cell cycle regulators (usually inhibitors). Furthermore, differential signaling by p38 has been demonstrated by the duration/strength of p38 activity, where transient activity leads to the proliferation of fibroblasts, but sustained signaling drives cell cycle arrest^46^. Our data clearly demonstrates that atypical p38 drives a spatiotemporal bias and so perturbing access to essential adaptors and transcription factors in the nucleus. Importantly, the p38 biosensor used in this platform detects spatial kinase activity and substrate availability. However, FRET responses are also a measure of phosphatase activity to control p38 activation and dephosphorylation of the sensor to terminate FRET signaling. While NaCl activity led to transient biosensor activation, demonstrating that FRET activation is reversible, thrombin activity was sustained for 30 min post-stimulation. Critically, SB203580 addition at 30 min demonstrated that the FRET sensor was not locked into an active conformation, triggering a decaying FRET response that confirms the presence of active phosphatases, and suggesting that p38 kinase activity is sustained to maintain a FRET response to 30 min. A specific phosphatase has not yet been identified for atypical p38 activity, and studies are ongoing to determine how atypical p38 signaling is terminated physiologically. Additional studies are also required to characterize the kinetics of dephosphorylation of activity reporters in models of endogenous GPCR expression. Defining temporal changes in phosphorylation and transcriptional control in the nucleus and cytosol will be essential for understanding the pathological triggers regulated by atypical p38 in vascular inflammation and cardiac damage.

In summary, we have successfully demonstrated a distinctive profile for spatiotemporal atypical p38 signaling by GPCRs. After the formation of the TAB1-p38 complex, TAB1 remains bound to p38, which is thought to spatially bias p38 from entering the nucleus or slow its translocation^8,10^. Indeed, p38-dependent phosphorylation of TAB1 has also been suggested to spatially bias TAB1 away from the nucleus into the cytosol^12,36^, and that over-expression of TAB1 can antagonize MKK3/6 activation, altering p38 subcellular localization^47^. Spatial restriction is an essential regulatory mechanism for many kinases. Our current data shed new light on these dynamics, demonstrating that although p38 activation by both MKK3/6 or TAB1 leads to the identical dual phosphorylation of p38, differential functional outcomes of atypical p38 are likely driven by limiting access to specific adaptors and transcription factors in the nucleus. Indeed TAB1-p38 activation is known to play essential roles in inflammation, including endothelial barrier homeostasis and activation of cytokine expression^10,11,48,49^. Spatial sequestration in the cytosol likely enhances access to these critical elements driving pathological signaling. However, further studies are required to define the specific downstream targets of atypical p38 and the specific mechanism that TAB1-p38 harness to regulate pathological signaling. Furthermore, Dyngo4A-dependent blockade of CME flipped the temporal dynamics of atypical p38 signaling, shifting the focus from endosomal and cytosolic to nuclear signaling responses. The current studies provide a framework by which to explore the spatiotemporal dynamics of atypical p38 signaling in GPCR-dependent and -independent mechanisms and screen for molecular regulators that could be therapeutically targeted. Future studies should focus on determining how the endosome selectively controls the spatial bias in atypical p38 activity and identifying the spatially restricted targets that drive pathological atypical p38 responses.

## Methods and materials

### Cell culture

HeLa cells (Cat. No. Hela CCl2, ATCC Manassa, VA, USA) were used for all experiments. Dulbecco’s modification of Eagle’s medium (DMEM, Cat. No. 10-013-CV, Corning, Mediatech Inc., Manassas, VA, USA) supplemented with 10% (v/v) fetal bovine serum (FBS; Cat. No. 35-010-CV, Corning, Mediatech, CA, USA) was used for cell maintenance. Cells were incubated at 37 °C in humidified conditions with 95% air and 5% CO_2_.

### Plasmids and cloning of spatial FRET biosensors

pcDNA3.1 FLAG-PAR1 was as previously described^9^. PTGER 2 and 4 (Prostaglandin E receptor 2 and 4) were a gift from Bryan Roth (Addgene plasmid #66484 and #66486, respectively. Each receptor subcloned from FLAG-PTGER2-TANGO and FLAG-PGTER4-TANGO, with a 3′ stop codon added to remove the TANGO tag^50^. The p38 FRET-NES (PerKy) biosensor was a kind gift from Dr. Saito (University of Tokyo, Japan) and has been previously characterized^18^. The FRET sensor was subcloned into pcDNA3.1 with spatial targeting motifs PCR-cloned onto the C-terminus of SECFP. The targeting motifs were (1) the plasma membrane, using a C-terminal lipid modification, Kras motif^19^, (2) the early endosome using 2xFVYE PI3P binding motif from mouse Hrs^20^, or the full-length wild-type Rab5^25^, (3) the nucleus using a nuclear localization signal motif^23^ and (4) diffuse cytosolic with nuclear export sequence^18^. mRFP-Rab5 was generated for this study using PCR-directed cloning. Briefly, mRFP (pcDNA3-mRFP was a gift from Doug Golenbock (Addgene plasmid # 13032; http://n2t.net/addgene:13032; RRID:Addgene_13032) was cloned onto the n-terminus of pcDNA3.1 Rab5 wt and Q79L^51,52^ using 5′ BamHI and 3′ EcoRI, sequences were verified using Sanger sequencing.

### Cell transfections

Cells were transfected in suspension with polyethyleneimine (PEI, Cat. No. 23966 polysciences) incubated with plasmid DNA in Gibco OptiMem Reduced Serum media (Cat. No. 31985-070, Life Technologies Corporation, NY, USA) before plating in 35 mm microscopy dishes (Cat. No. 81156, Ibidi, Gräfelfing, Germany) coated with human fibronectin, 1.5 µg/cm^2^ (Cat No. 356008 Corning, Mediatech Inc., Manassas, VA, USA) with DMEM (10% FBS) in a humidified incubator for 24 or 48 h.

### Chemicals and reagents

Starvation medium composed of 0.1% bovine serum albumin (BSA, Cat. No. BP1600-100, Fisher bioreagents, NJ, USA) 20 mM HEPES (Cat. No. H3537, Sigma-Aldrich, MA, USA) 1 mM CaCl (Cat. No. BDH9224, BDH Chemicals, OH, USA) in phenol-red free DMEM (Cat. No. SH30284.01, HyClone, UT, USA) and filtered through a nylon 0.45uM filter, was applied to transfected cells for 24 h before live-cell imaging and exchanged to FRET imaging buffer (20 mM HEPES, 1 mM CaCl_2_, 2 g/L d-glucose (158968-500G, Sigma-Aldrich, MO, USA) 0.23 mM Sodium Pyruvate (SH30239.01, HyClone, UT, USA) in Gibco Hanks Modified Balanced Salt Solution (Cat. No. 14065-056, Life Technologies Corporation, NY, USA), pH 7.4. SB203580, (Cat. No. S-3400, LC Laboratories, MA, USA, used at 25 µM for 1 h), Dynamin Dyngo 4A (Cat. No. ab120689, Abcam, Cambridge, UK, used at 15 µM, 1 h.). Rabbit anti-EEA1 (Cat No. C45B10 Cell signal Technologies), rabbit-anti-GAPDH-Alexa647 (Cat No. 3907, Cell signaling technologies), rabbit Alexa fluor-594 (Cat No. A11012. Fisher Scientific). Rabbit anti-total p38 (Cat No. 9212, Cell signal Technologies), Rabbit anti-phospho-p38 (Cat No. 4511, Cell signal Technologies), HRP-conjugated goat–anti-rabbit (Cat No. Bio-Rad Laboratories. Biotracker 555 Orange cytoplasmic membrane dye (Cat No. SCT107, EMD Millipore Corp). RedDot^TM^2-far-red nuclear stain (Cat No. 40060, Biotium).

### Fluorescence resonance energy transfer (FRET)-based live cell imaging

Images for live-cell fluorescence assays were acquired using a Zeiss LSM-800 microscope (Zeiss, Jena, Germany), Colibri LED, Axiocam 506 mono, and Plan-Apochromat 20x/0.8 M27 objective, with a heated environment stage 37 °C in humidified conditions with 95% air and 5% CO_2_. An excitation wavelength of 433 nm, emission 475 nm filter, was used for “SECFP”, while an excitation wavelength of 433 nm, emission 524 nm filter was used for “FRET”. Channel images were acquired sequentially with 60 ms exposure for each channel. Image collection used Zeiss Zen Blue 3.0 software. Each channel was background corrected, and the pixel-by-pixel brightness ratio of the “FRET” channel to the “SECFP” channel within each region of interest (ROI) was calculated. FRET/SECFP channel light intensity was adjusted prior to data collection to achieve a baseline ratio average of 1.0. Typically, between thirty and fifty individual whole-cell ROIs were taken per experiment. In addition, ROIs were mapped across the experiment to follow cell movement. Cell exclusion criteria were limited to cells with a saturated SECFP or FRET intensity, cells morphologically indicative of death/division (rounded up or blebbing), or if cells were not wholly in the imaging frame. Images were collected using epifluorescence microscopy and presented as a ratiometric analysis of the emission from cyan (SECFP) and yellow (YFP/FRET). Fluorescence intensities were background-corrected by subtracting the background fluorescence intensity of a cell-free region from the emission intensities of biosensor-expressing cells. As such the FRET ratio from a small area versus the whole cell is comparable as the FRET response is normalized to the total CFP in the ROI rather than a fraction of the total area. As such an endosome/PM versus the whole cell ROI yields comparable dynamic responses (Supp. Fig. 13). Cells were acclimated in the heated environment for 10 min prior to imaging. Cells were incubated for 5 min or until fluorescent signals stabilized before stimulation. Dyngo4A-treated trials were performed in parallel to non-treated trials to ensure experimental comparability between conditions.

For high-resolution 63× confocal imaging, live-cell images were collected with a 0.4 µm z-section. FRET sensor expressing cells were co-labeled with RedDot2 (1:200) at 37 °C for 20 min, media was exchanged with phenol red free DMEM before live-cell imaging. For PM biotracker orange, cells were placed at 4 °C and stained with 5 µl per ml for 15 min. Cells were then rinsed with phenol red free DMEM before being fixed with 4% paraformaldehyde, washed with PBS and immediately imaged, as slide mounting disrupted PM labelling. For co-labeling with EEA1 or GAPDH-647, transfected cells were fixed with 4% paraformaldehyde, permeabilized with 0.1% in triton X100, then incubated overnight at 4 °C with a primary rabbit anti-EEA1 (1:250) antibody and secondary anti-rabbit Alexa-596 (1:500), or GAPDH-647 (1:250) mounted and imaged with a plan-apochromat 63×/1.4 oil DIC M27 objective. Pearson Correlation Coefficient (*r*) was calculated within Zen Blue 3.0 software from whole cell ROIs.

### Immunoblotting

HeLa cells were seeded into 24-well plates, transfected, and grown as described above. Cells were serum starved overnight at 37 °C before agonist stimulation with 10 nM thrombin or 10 µM PGE2 as previously described^9,10^. Cells lysed in 1X Laemmli sample buffer plus 100 mM DTT, boiled for 2 minutes and vortexed, cell lysates resolved by SDS-PAGE, and processed for immunoblotting as previously described^10^, with antibody dilutions of 1:4000 for total p38 and 1:3,500 for phospho-p38. Immunoblots were quantified by densitometry using NIH ImageJ software.

### Data analysis

All data were analyzed using Prism 9.0 software (GraphPad Software, La Jolla, CA) to unbiasedly calculate FRET max and T1/2 values. Prism was also used to calculate statistical significance using Student’s *t-test* and one-way  analysis of variance (ANOVA), as indicated.

## Supplementary Information

Supplementary Figures.

## Data Availability

All datasets used and/or analyzed during the current study are available from the corresponding author upon reasonable request.

## Abbreviations

ANOVA: Analysis of variance
FRET: Fluorescence resonance energy transfer
GPCR: G protein-coupled receptor
MAPK: Mitogen-activated protein kinase
MKK3/6: Mitogen-activated protein kinase kinase 3/6
PAR1: Protease-activated receptor-1
PGE2: Prostaglandin E2
TAB1: Transforming growth factor-β activated kinase-1 binding protein1
α-Th: Thrombin
