# Supplementary material for: Fluorescence resonance energy transfer (FRET) spatiotemporal mapping of atypical P38 reveals an endosomal and cytosolic spatial bias
Source: Sci Rep. 2023 May 8;13:7477. doi: 10.1038/s41598-023-33953-y (PMC10167256; doi:10.1038/s41598-023-33953-y)

Fluorescence Resonance Energy Transfer (FRET) Spatiotemporal Mapping of Atypical P38 Reveals an Endosomal and Cytosolic Spatial Bias

Jeremy C. Burton[1], Jennifer Okalova[1,2], and Neil J. Grimsey[1, *]

Supplemental Figures

Supplemental Figures

Supplemental Figure 1: p38 Biosensors Colocalize With Compartment-Specific Probes And Proteins
Immunofluorescence confocal microscopy of fixed HeLa cells transiently expressing localized biosensors (displaying-SECFP) and probed for subcellular-specific markers (as indicted) as follows: **A)** plasma membrane with PM-Orange **B)** cytosolic with GAPDH-Alexa647 **C)** nucleus with Nuc-red **D)** 2XFYVE endosomal with wt-RAB5-mRFP **E)** 2XFYVE endosomal co-transfected with Q79L-RAB5-mRFP **F)** RAB5 endosomal with EEA1(Alexa 594) **G)** Pearson's Correlation Coefficient (*r*) quantifying biosensor-protein colocalization as described in **A-F**. n = number of individual cells, representative of >2 independent biological repeats. Scale bars, 10 μm.

Supplemental Figure 2: Pseudo Color Images Of Biosensor Responses To NaCl
Representative FRET/SECFP ratiometric images (pseudo colored) before and after treatment with NaCl, cells expressing **A)** NES - cytosolic **B)** NLS - nuclear **C)** KRAS - plasma membrane **D)** 2XFYVE - endosome or **E)** RAB5 - endosome. Look Up Table (LUT) pseudo coloring where warmer colors represent higher FRET emission ratios and indicate spatially localized activity. Scale bars, 10 μm.

Supplemental Figure 3: Pseudo Color Images Of Biosensor Responses To Thrombin
Representative FRET/SECFP ratiometric images (pseudo colored) before and after treatment with thrombin, cells expressing **A)** NES - cytosolic **B)** NLS - nuclear **C)** KRAS - plasma membrane **D)** 2XFYVE - endosome or **E)** RAB5 - endosome. Look Up Table (LUT) pseudo coloring where warmer colors represent higher FRET emission ratios and indicate spatially localized activity. Scale bars, 10 μm.

Supplemental Figure 4: Thrombin-induced p38 Activity Is Not Altered By Addition Of DMSO Vehicle Control
Activation of p38 using FRET biosensors localized to the **A)** plasma membrane **B)** cytosol **C)** nucleus or **D)** endosome, expressed in HeLa cells incubated with 10nM thrombin and vehicle DMSO. FRET ratios were normalized prior to thrombin addition. Representative normalized average FRET ratio, n > 20 cells +/- SEM, two independent biological repeats per biosensor.

Supplemental Figure 5: RAB5 Biosensor Endosomal Signaling By NaCl And Thrombin
Activation of p38 using wt-RAB5 endosomal biosensor expressed in HeLa cells incubated with **A)** thrombin 10 nM **B)** NaCl 300 mM. **C)** SB203580 25 μM preincubation and thrombin, or **D)** SB203580 25 μM preincubation and NaCl. Representative FRET ratios were normalized prior to agonist addition. Individual cell FRET ratios are depicted in gray (n > 30-60 cells per biosensor). Average FRET ratio is indicated by colored lines.

Supplemental Figure 6: EP2 Induced p38 Activation

Activation of p38 using FRET biosensors localized to the plasma membrane **A)** cytosolic **B)** nuclear **C)** or endosomal **D)** expressed in HeLa cells co-expressing EP2, incubated with 10 μM PGE2. FRET ratios were normalized prior to PGE2 addition. Individual cell FRET ratios are depicted in gray (n > 30-60 cells per biosensor). The average FRET ratio is indicated by colored lines. **E)** Overlay of representative normalized averages of FRET ratio in biosensor platform from **A-D)**. **F)** Maximum change in FRET ratio ($FRET_{MAX}$) of individual cells pooled from 3 independent repeats, (n > 80-150 ROIs per biosensor. mean +/- SEM) were analyzed by One-Way ANOVA (****, $p < 0.0001$). G**)** FRET response kinetics, T ½ values of cells in A-D (mean +/- SEM).

Supplemental Figure 7: EP4 Induced p38 Activation

Activation of p38 using FRET biosensors localized to the plasma membrane **A)** cytosolic **B)** nuclear **C)** or endosomal **D)** expressed in HeLa cells co-expressing EP4, incubated with 10 μM PGE2. FRET ratios were normalized prior to PGE2 addition. Individual cell FRET ratios are depicted in gray (n > 30-60 cells per biosensor). The average FRET ratio is indicated by colored lines. **E)** Overlay of representative normalized averages of FRET ratio in biosensor platform from **A-D)**. F) Maximum change in FRET ratio ($FRET_{MAX}$) of individual cells pooled from 3 independent repeats, (n > 80-150 ROIs per biosensor. mean +/- SEM) were analyzed by One-Way ANOVA (****, $p < 0.0001$). G**)** FRET response kinetics, T ½ values of cells in A-D (mean +/- SEM).

Supplemental Figure 8: p38 Biosensor is Specific to Kinase Activity

A) Schematic of biosensor design for FRET-Null, with key serine residues in p38 bait substrate mutated to alanine. Null-biosensor expressed in HeLa cells incubated with, **A)** thrombin 10nM **B)** NaCl 300 mM. Representative FRET ratios were normalized prior to agonist addition. Individual cell FRET ratios are depicted in gray (n > 30-60 cells per biosensor). The average FRET ratio is indicated by colored lines.

Supplemental Figure 9: Dyngo Treatment Does Not Alter Signaling Dynamics of Osmotic Stress

Activation of p38 using FRET biosensors localized to the plasma membrane **A)** cytosolic **B)** nuclear **C)** or endosomal **D)** expressed in HeLa cells incubated with 300 mM NaCl. FRET ratios were normalized prior to NaCl addition. Individual cell FRET ratios are depicted in gray (n > 30-60 cells per biosensor). The average FRET ratio is indicated by colored lines. **F)** Overlay of representative normalized averages of FRET ratio in biosensor platform from **B-E)**. **G)** Maximum change in FRET ratio ($FRET_{MAX}$) of individual cells pooled from 3 independent repeats, (n > 80-150 ROIs per biosensor. mean +/- SEM) were analyzed by One-Way ANOVA (****, $p < 0.0001$). **H)** FRET response kinetics, T ½ values of cells in A-D (mean +/- SEM).

Supplemental Figure 10: Dyngo Treatment Shifts p38 Activity Away From The Cytosol And Endosome

Activation of p38 using FRET biosensors localized to the plasma membrane **A)** cytosolic **B)** nuclear **C)** or endosomal **D)** expressed in HeLa cells co-expressing PAR1, incubated with 10 nM thrombin. FRET ratios were normalized prior to thrombin addition. Individual cell FRET ratios are depicted in gray (n > 30-60 cells per biosensor). The average FRET ratio is indicated by colored lines. **E)** Overlay of representative normalized

averages of FRET ratio in biosensor platform from **A-D)**. **F)** Maximum change in FRET ratio ($FRET_{MAX}$) of individual cells pooled from 3 independent repeats, (n > 80-150 ROIs per biosensor. mean +/- SEM) were analyzed by One-Way ANOVA (****, $p < 0.0001$). **G)** FRET response kinetics, T ½ values of cells in A-D (mean +/- SEM).

Supplemental Figure 11: Dyngo Treatment Does Not Inhibit P38 Activation
A) Representative immunoblot of HeLa cells transiently expressing PAR1 and stimulated with NaCl 300 mM or 10 nM thrombin for the indicated times in the presence of 15µM Dyngo-4A or DMSO control. Fold change of phospho-p38 over total p38 quantified from three independent repeats. (mean +/- StDev). **B)** Unprocessed chemiluminescent image of blot from A), merged with colorimetric image to show molecular weight marker.

Supplemental Figure 12: Dyngo Treatment RAB5 Signaling
Activation of p38 using wt-RAB5 endosomal biosensor expressed in HeLa cells incubated with **A)** cells preincubated with Dyngo 4A (15 µM) for 1h prior to stimulation with thrombin 10nM **B)** NaCl 300 mM. SB203580 added at 30 minutes of stimulation as indicated. Representative FRET ratios were normalized prior to agonist addition. Individual cell FRET ratios are depicted in gray (n > 30-60 cells per biosensor). Average FRET ratio is indicated by colored lines.

Supplemental Figure 13: Comparable FRET Responses From Whole Cell or Endosomal Puncta
Representative FRET/SECFP channel emission ratio time course comparing responses from an ROI drawn around a whole cell (in blue) and an ROI drawn around a single endosome (in purple) in HeLa cells transfected with pcDNA3.1 FLAG-PAR1 and 2XFYVE endosome localized biosensor, that endosomal p38 activity responses specifically come from endosomes. Representative fluorescent (merged YFP/SECFP) Colibri image. Scale bar 10 µM.

Supplemental Figure 1

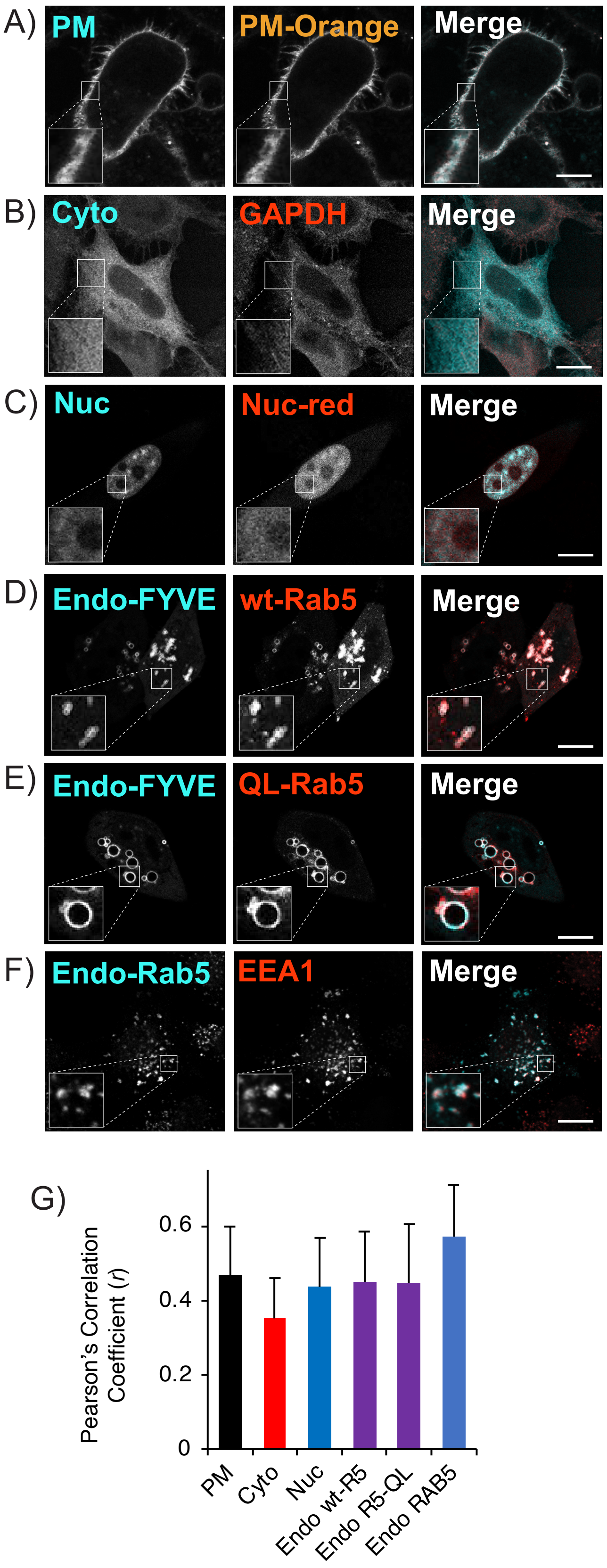

Supplemental Figure 2

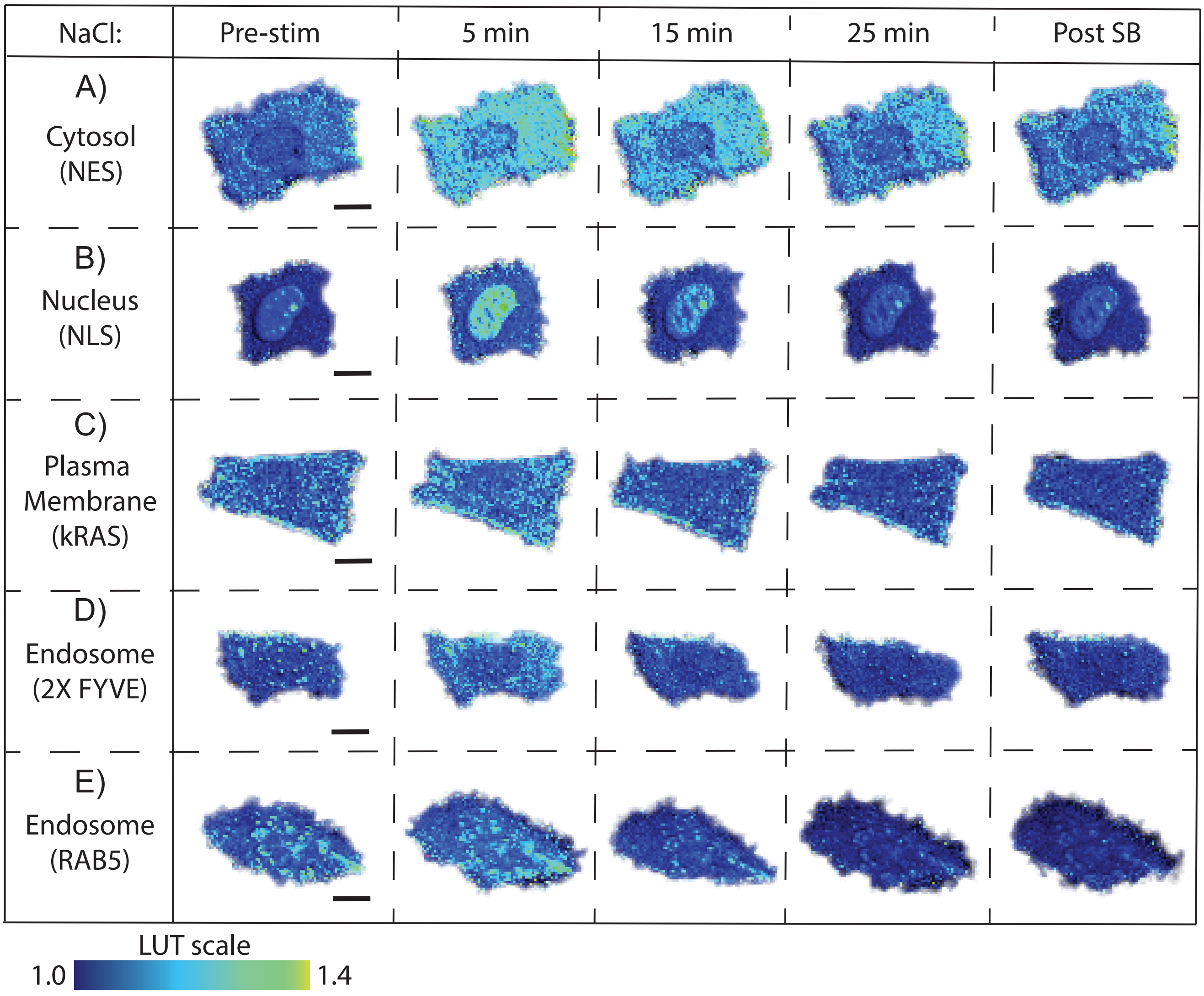

Supplemental Figure 3

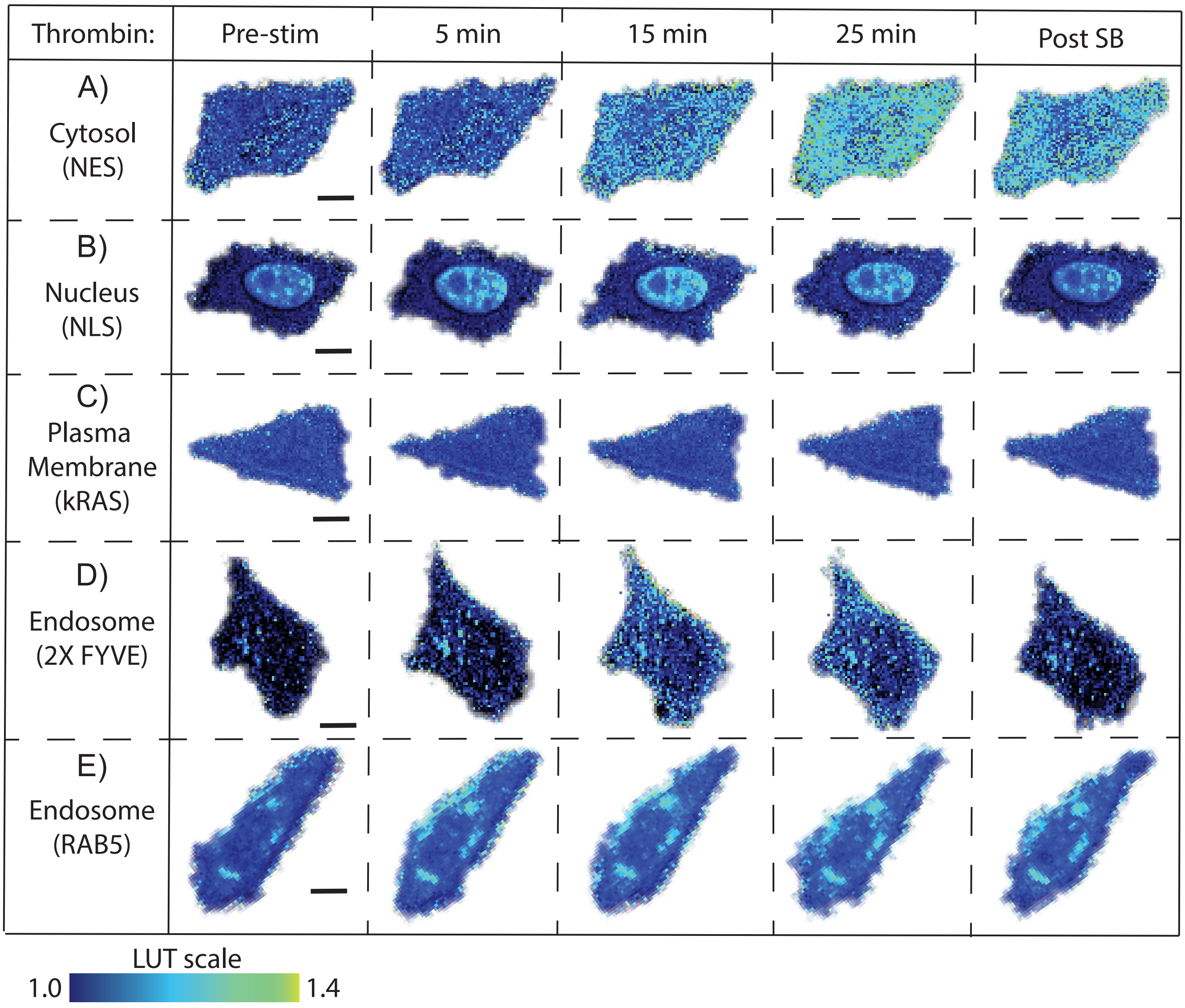

Supplemental Figure 4

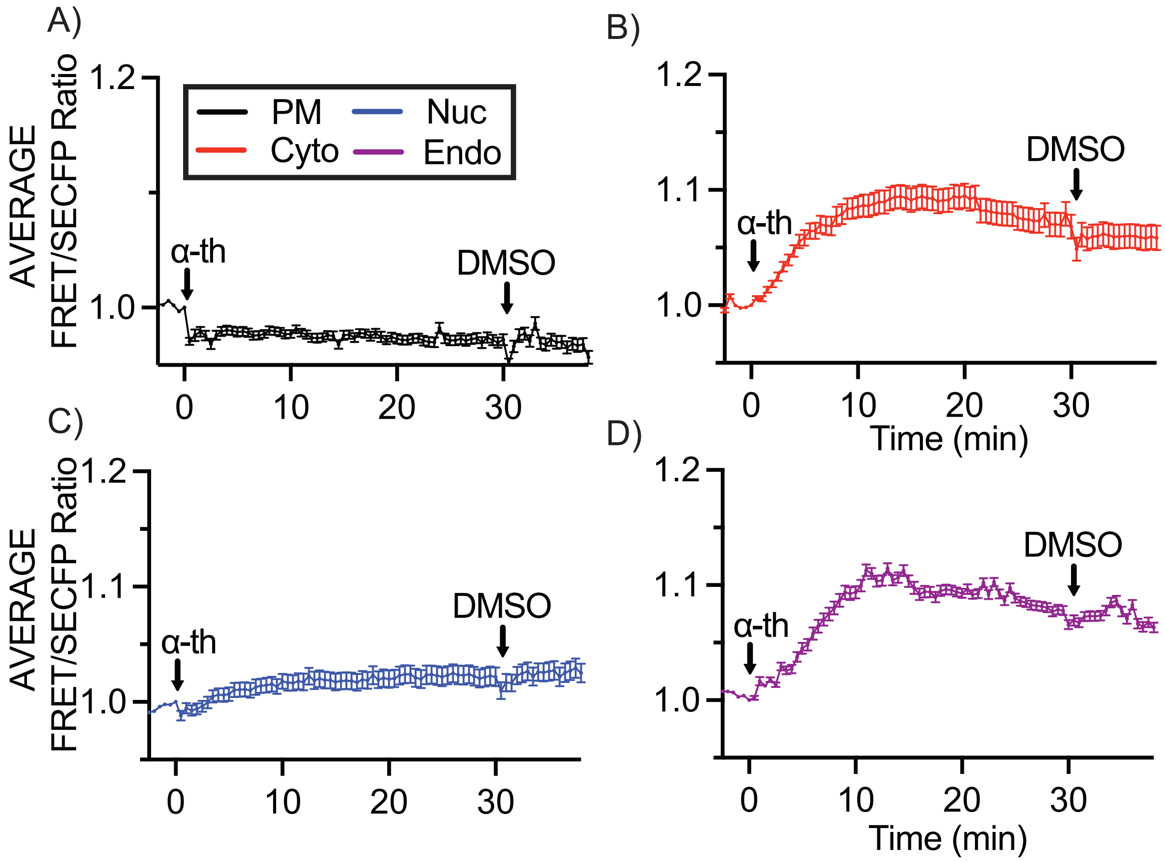

Supplemental Figure 5

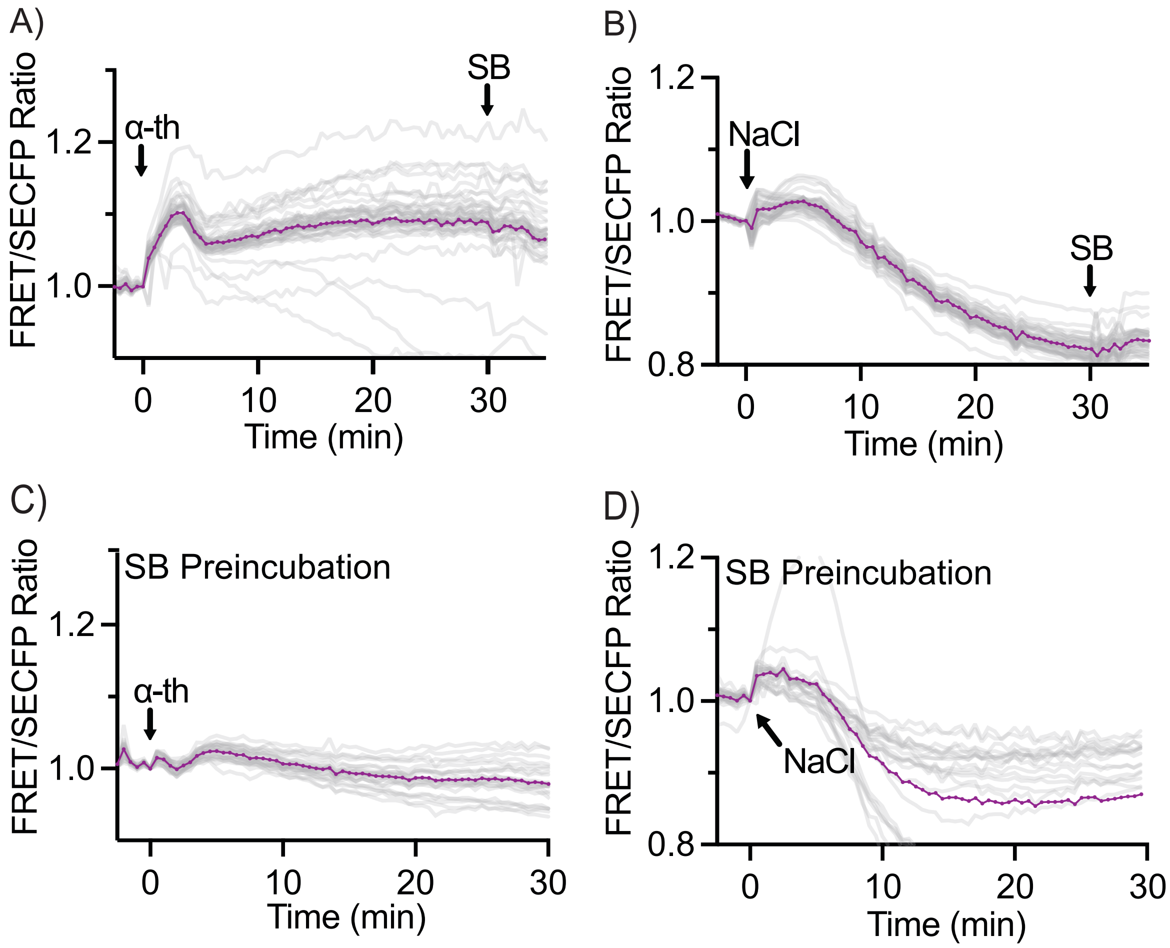

Supplemental Figure 6

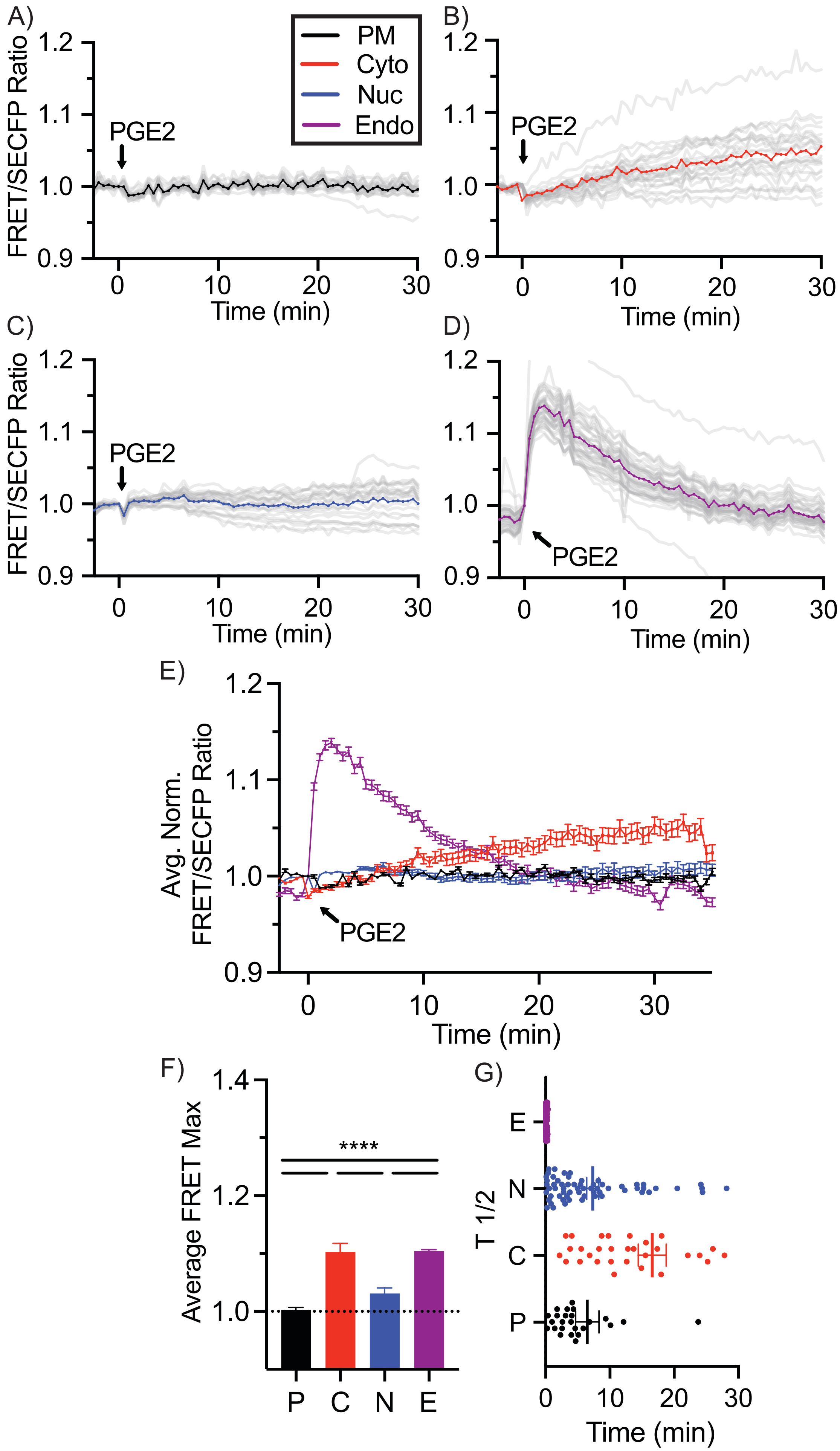

Supplemental Figure 7

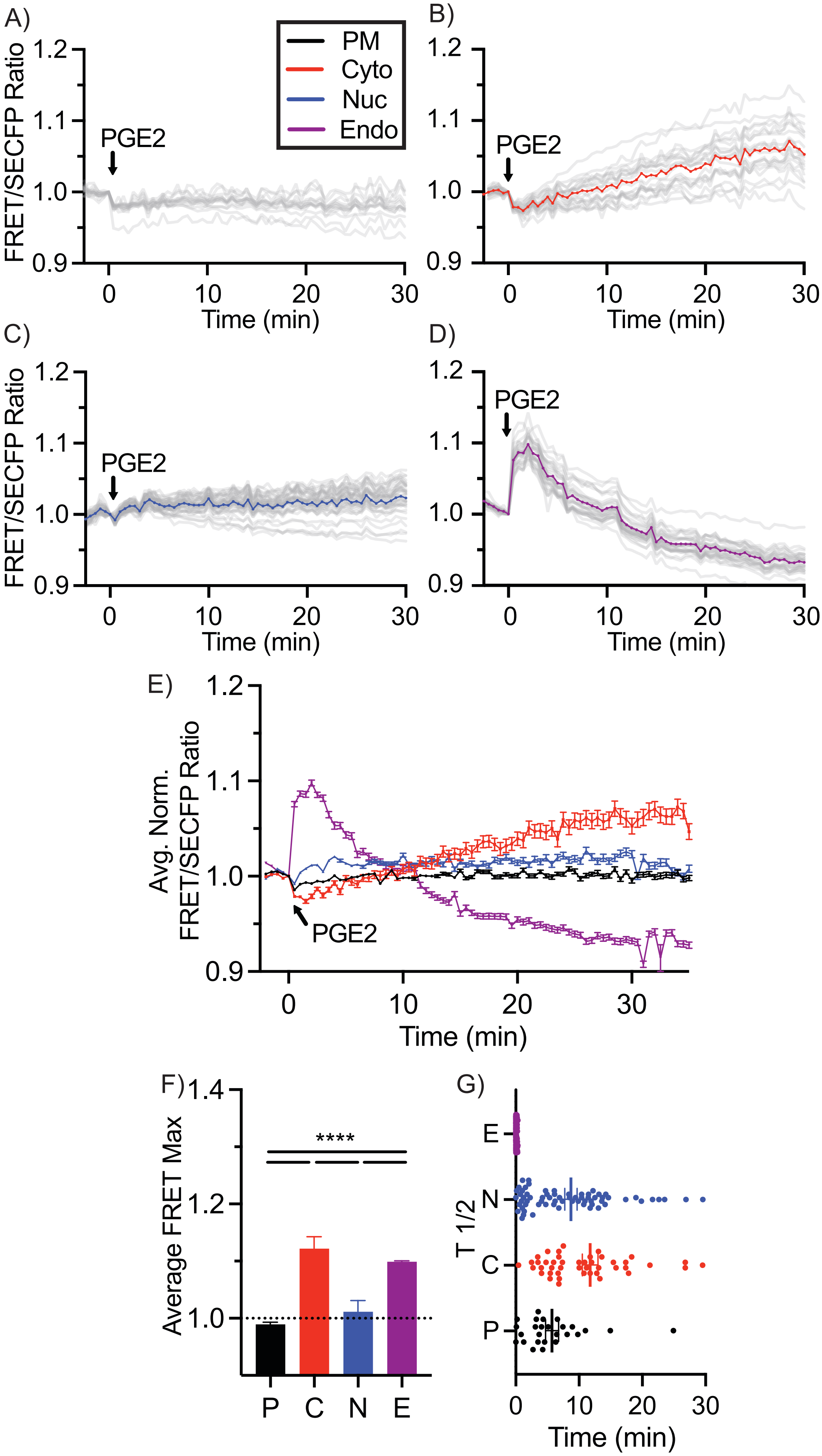

Supplemental Figure 8

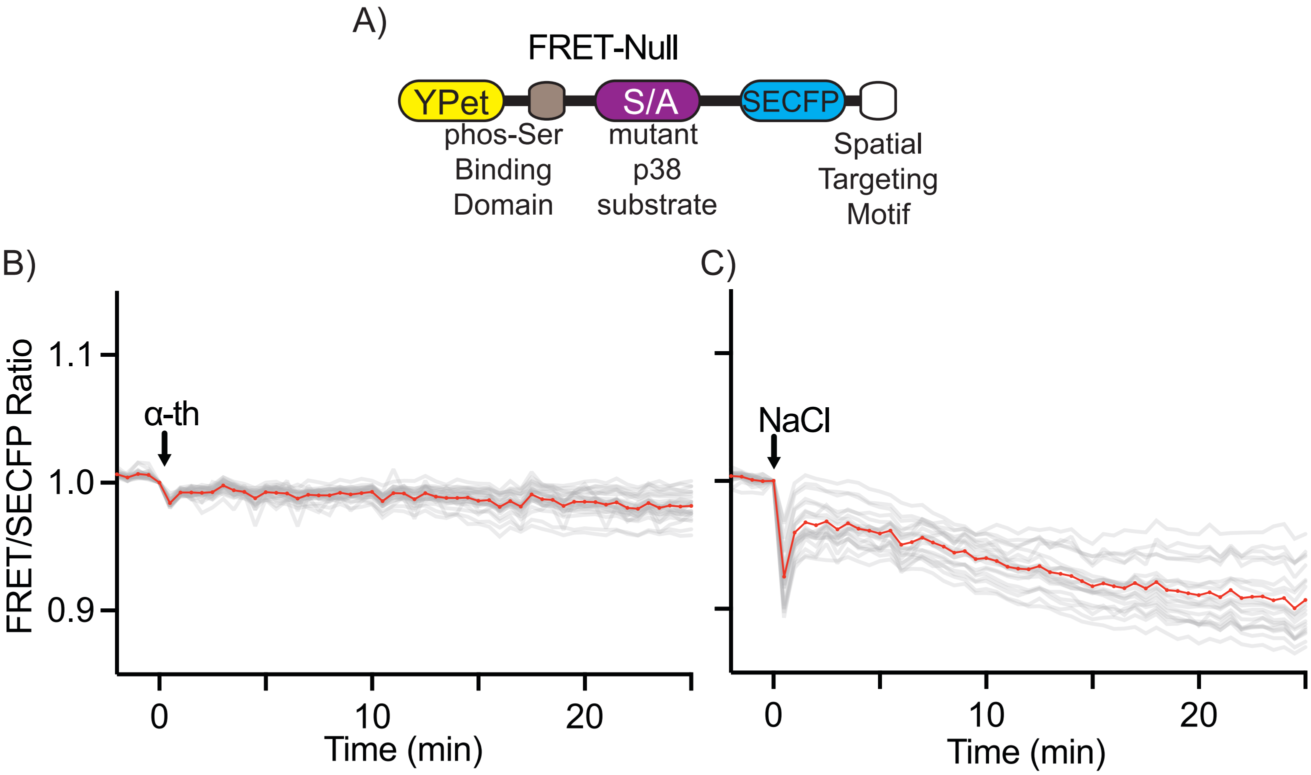

Supplemental Figure 9

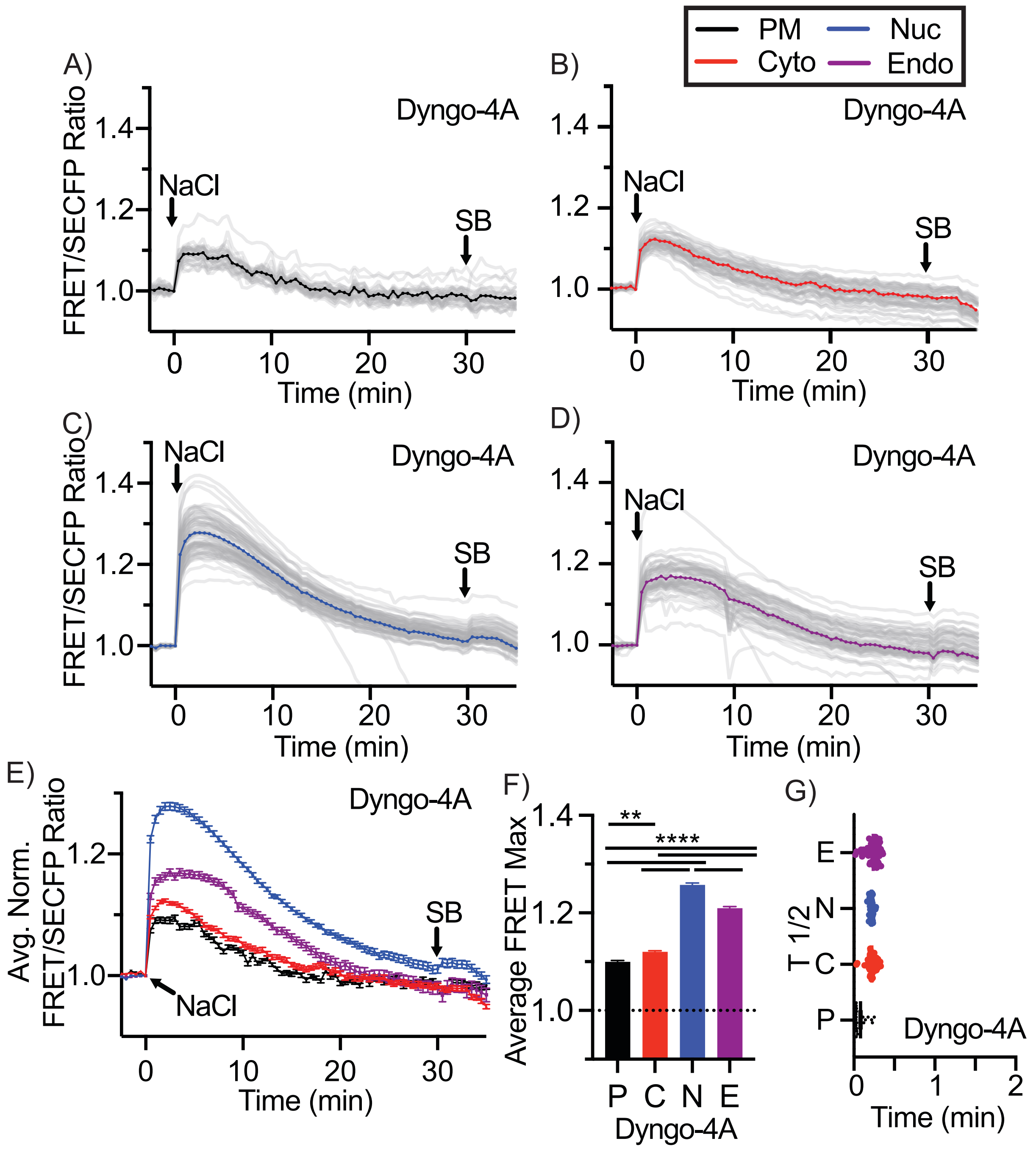

Supplemental Figure 10

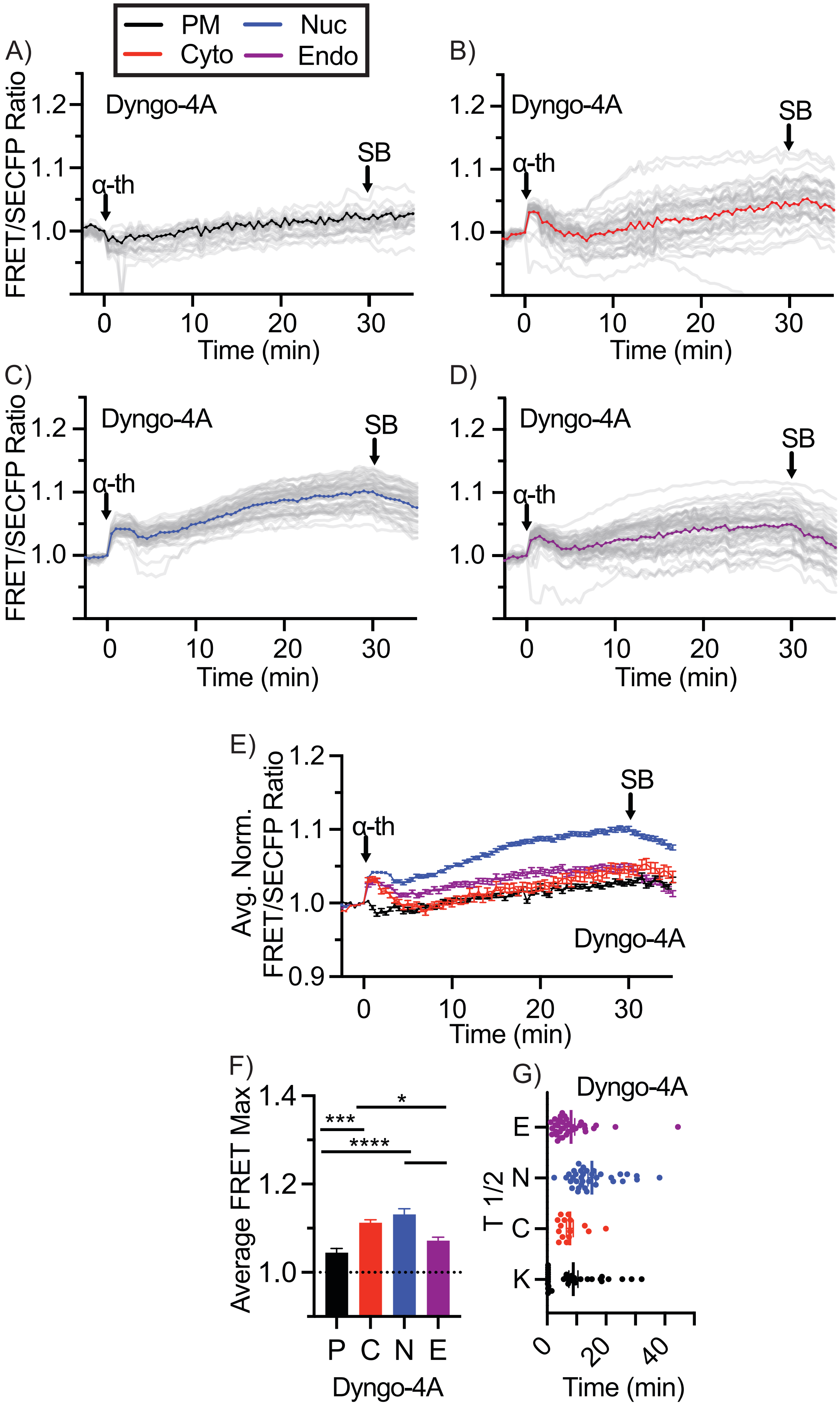

Supplemental Figure 11

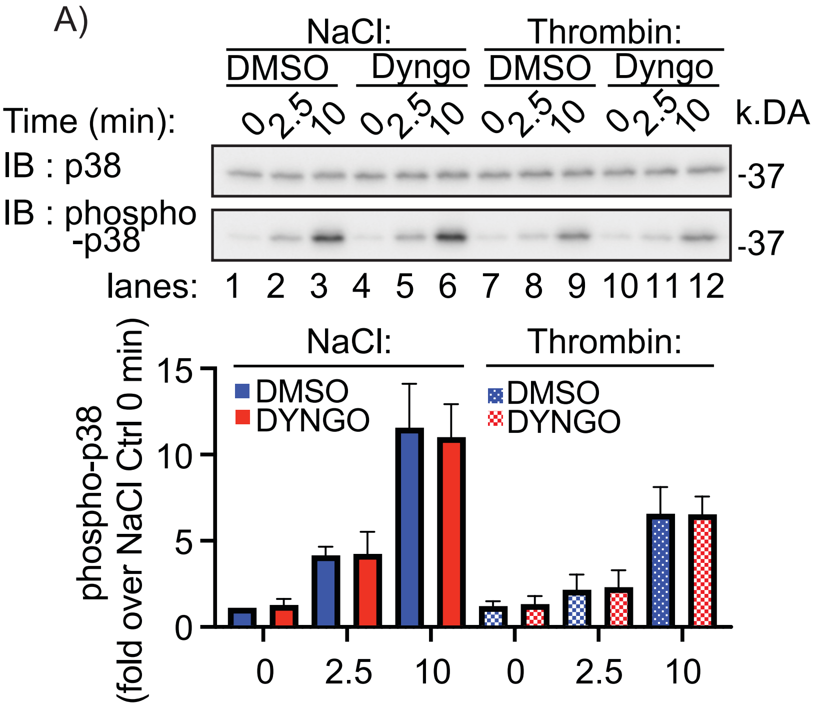

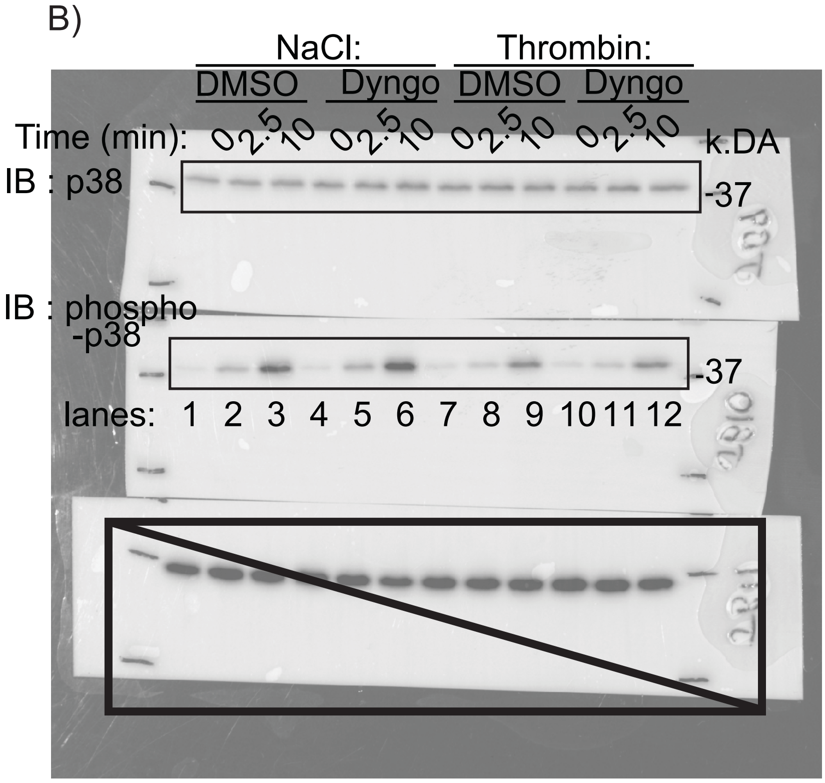

Merged chemiluminescent and colorimetric images

Supplemental Figure 12

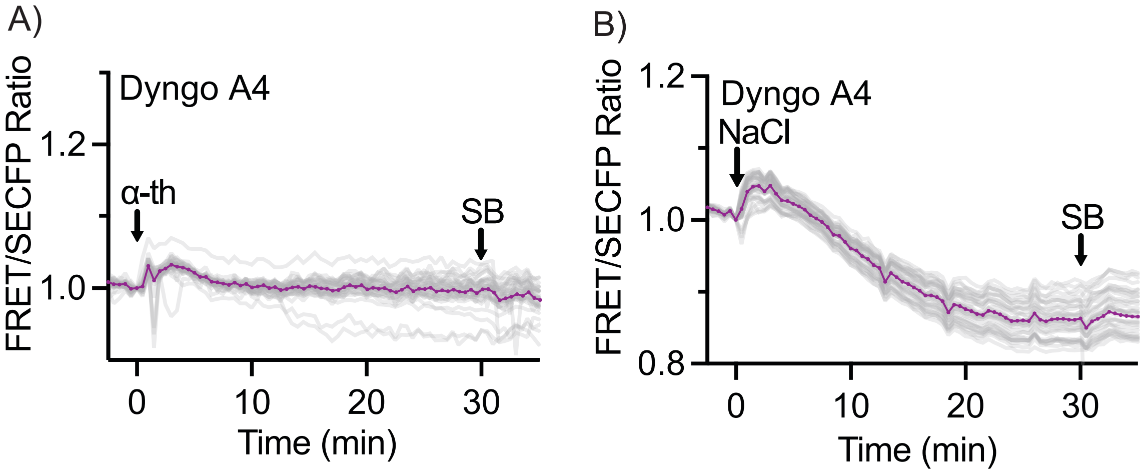

Supplemental Figure 13

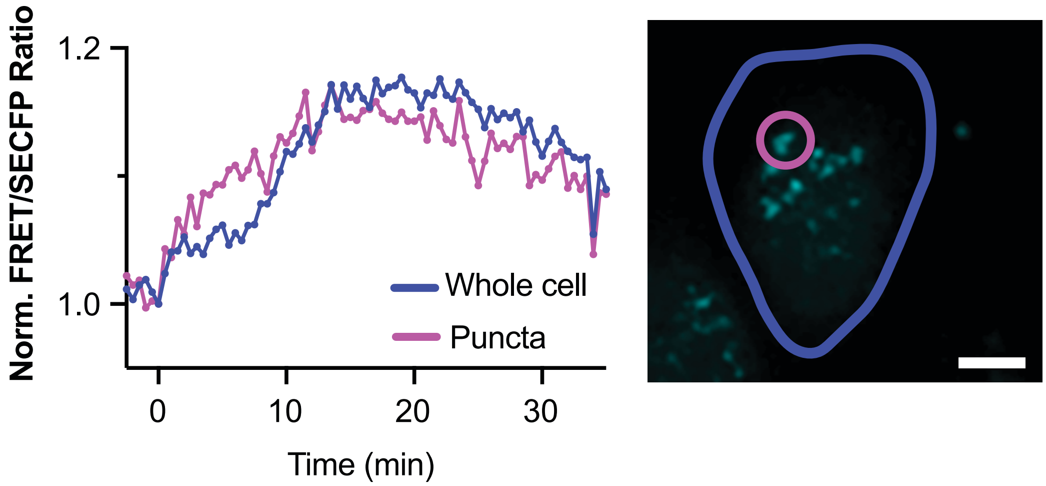

Supplement: Supplementary file 1 — Supplementary Figures. [file 41598_2023_33953_MOESM1_ESM.pdf]
